# Antiplasmodial peptaibols act through membrane directed mechanisms

**DOI:** 10.1016/j.chembiol.2023.10.025

**Published:** 2024-02-15

**Authors:** Jennifer E. Collins, Jin Woo Lee, Frances Rocamora, Gagandeep S. Saggu, Karen L. Wendt, Charisse Flerida A. Pasaje, Sebastian Smick, Natalia Mojica Santos, Raphaella Paes, Tiantian Jiang, Nimisha Mittal, Madeline R. Luth, Taylor Chin, Howard Chang, James L. McLellan, Beatriz Morales-Hernandez, Kirsten K. Hanson, Jacquin C. Niles, Sanjay A. Desai, Elizabeth A. Winzeler, Robert H. Cichewicz, Debopam Chakrabarti

**Affiliations:** 1 Burnett School of Biomedical Sciences, University of Central Florida, Orlando, FL 32826, USA; 2 Department of Chemistry and Biochemistry, Institute for Natural Products Applications & Research Technologies, University of Oklahoma, Norman OK 73019, USA; 3 Division of Host-Microbe Systems & Therapeutics, Department of Pediatrics, University of California San Diego, La Jolla, CA 92093, USA; 4 Laboratory of Malaria and Vector Research, National Institute of Allergy and Infectious Diseases, NIH, Rockville, MD 20852, USA; 5 Department of Biological Engineering, Massachusetts Institute of Technology, Cambridge, MA 02142, USA; 6 Department of Molecular Microbiology and Immunology and South Texas Center for Emerging Infectious Diseases, University of Texas San Antonio, San Antonio, TX 78249, USA

**Keywords:** peptaibol, *Plasmodium*, malaria, MDR1, digestive vacuole, antiplasmodial, ion channel, peptides, AMPs, Alamethicin

## Abstract

Our previous study identified 52 antiplasmodial peptaibols isolated from fungi. To understand their antiplasmodial mechanism of action, we conducted phenotypic assays, assessed the *in vitro* evolution of resistance, and performed a transcriptome analysis of the most potent peptaibol, HZ NPDG-I. HZ NPDG-I and 2 additional peptaibols were compared for their killing action and stage dependency, each showing a loss of digestive vacuole (DV) content via ultrastructural analysis. HZ NPDG-I demonstrated a stepwise increase in DV pH, impaired DV membrane permeability, and the ability to form ion channels upon reconstitution in planar membranes. This compound showed no signs of cross resistance to targets of current clinical candidates, and 3 independent lines evolved to resist HZ NPDG-I acquired nonsynonymous changes in the *P. falciparum* multidrug resistance transporter, pfmdr1. Conditional knockdown of PfMDR1 showed varying effects to other peptaibol analogs, suggesting differing sensitivity.

## Highlights

• Peptaibol properties impact hemolysis, cytotoxicity, and antiplasmodial activity
• All peptaibols tested demonstrate digestive vacuole disruption as a commonality
• The most potent peptaibol, HZ NPDG-I, shows sensitivity to PfMDR1 knockdown

## Graphical abstract

**Figure:**
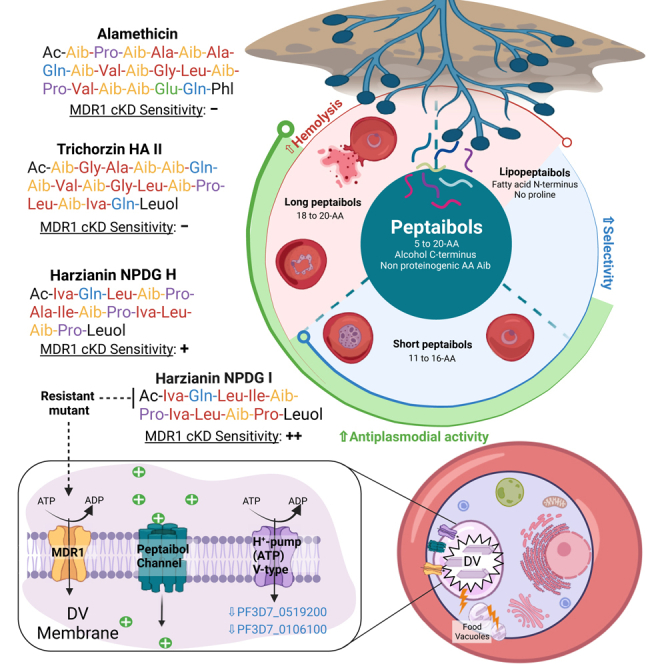

## Teaser

In the present study, Collins et al. explore peptaibols and their potential against the malaria parasite. These unique and promising compounds show variable effects on the parasite’s life cycle, with key shared features that might point toward a membrane-centric mode of action.

## Introduction

Malaria, caused by the apicomplexan parasite *Plasmodium*, was responsible for over 619,000 reported deaths in 2022, an increase from 568,000 deaths pre-pandemic.^1^ This disease represents a global threat to health and economic stability and is currently exacerbated by the propagation of drug resistance.^2^^,^^3^^,^^4^^,^^5^^,^^6^ As a result, new treatments and therapeutic targets are needed. A unique class of natural products that may meet this requisite is peptaibols. These and other peptide-based drugs are highly distinct from traditional small molecules and offer improved selectivity and minimal side effects.^7^^,^^8^ Interest in these drugs has grown in recent years, with over a hundred peptide drugs in clinical trials in 2020.^9^^,^^10^ However, their therapeutic promise is somewhat hampered by various liabilities such as toxicity,^11^ proteolytic degradation, and poor bioavailability.^12^ While more traditional antimicrobial peptides (AMPs) have received some attention in *Plasmodium* as both therapeutics^13^^,^^14^ and molecular probes,^15^^,^^16^ little if any attention has been given to the unique subclass of peptaibols.^17^

Peptaibols are a series of compounds named for being peptides that contain the non-standard amino acid Aib (α-aminoisobutyric acid) and an alcohol on their *C*-terminus. Aib is a strong helix promoter, predisposing these compounds to the arrangement of helical bundles in biological membranes. Unlike other AMPs, peptaibols lack most charged and polar amino acids.^18^ To date, more than 440 peptaibols have been identified, ranging from 5 to 20-AAs in length.^19^ They can be categorized as “long” (18-20-AAs), “short” (11-16-AAs), and “lipopeptaibols,” which contain a fatty acid *N*-terminus.^19^^,^^20^ Peptaibols with 17-AAs were originally not included in these categories but have since been identified.^21^^,^^22^ Longer, even numbered peptaibols predominate, likely due to the group’s transmembrane spanning functionality.^19^ The notable exception being the short and odd numbered 11-AA peptaibols, which make up a sizable distribution. This length is likely significant, as it corresponds to the approximate distance of a half-bilayer spanning helix.^18^

Peptaibol compounds display a wide range of activities; including antifungal,^23^^,^^24^ antibacterial,^25^^,^^26^ anticancer,^27^ and antiparasitic.^28^ Their potency is heavily linked to their capacity to permeabilize lipid bilayers.^19^ Three models are used to describe this action on a mechanistic level. The first is the “barrel stave” model, wherein (1) peptaibols bind to the membrane as monomers or multimers, (2) peptaibols in a membrane-bound state recognize each other, (3) they insert into the lipid bilayer, and (4) they aggregate into a barrel-like structure, potentially recruiting additional monomers and increasing pore diameter.^29^^,^^30^ A second method is outlined by the “carpet” model, where (1) peptaibols bind to the surface of a membrane until (2) a critical threshold is reached, and (3) membrane permeation occurs from bilayer disruption.^29^^,^^30^ Finally, there is the “toroidal pore” model in which (1) peptaibols aggregate and attach to the membrane then (2) induce the lipid monolayer to bend around them, forming a pore lined with peptaibol and lipid head groups.^31^ One or more of these mechanisms may predominate depending on the peptaibol.^29^^,^^30^^,^^32^ The formation of barrel stave voltage-gated ion channels is typically associated with long peptaibols able to span a bilayer.^22^^,^^33^ The 20-AA peptaibol alamethicin is a prime example of this and is widely used as the canonical model for peptaibol ion channel formation.^34^ Despite this, short peptaibols have been found that exhibit pronounced membrane activity on par with their longer counterparts.^32^ It has been suggested these short peptaibols function either by doubling up and joining end-to-end within the bilayer, by bending the membrane to form toroidal channels, or by acting in a detergent-like manner.^18^ The mechanism utilized depends both on the properties of the peptaibol (length, AA residues, hydrophobicity) and the biological environment of the membrane in question (pH, lipid content, etc.).^31^ How these factors may influence peptaibol behavior in *Plasmodium* is currently unclear.

Herein, we investigated the mechanisms that influence the activity of 57 distinct peptaibols, including long peptaibols (18-AA and 20-AA), short peptaibols (14-AA and11-AA), and lipopeptaibols (7-AA, 11-AA, and 15-AA). We define features that promote antiplasmodial potency and selectivity, and those that reduce hemolysis, one of the major factors responsible for peptide toxicity.^10^ Additionally, we explore the *in vitro* antiplasmodial mechanisms of a subset of peptaibols, with an emphasis on their impact to the parasite digestive vacuole (DV).

## Results

### Peptaibol properties influence antiplasmodial potency, hemolysis, and selectivity

As peptaibols are prominent pore-forming molecules, we tested their potential for red blood cell (RBC) hemolysis. All 52 peptaibols and lipopeptaibols identified in our original publication^35^ were assessed, in addition to alamethicin. Following the previous publication, 4 new isomers of the 11-AA peptaibols, 2 diastereomers, and 2 amides were synthesized. Their activity remained at—or near—their original counterparts, with the exception of the harzianin HB I amide, which showed decreased inhibition. We included these 4 isomers in our hemolysis assay and have provided their activity, spectra, and purity information in Data S1.

**Figure 1:**
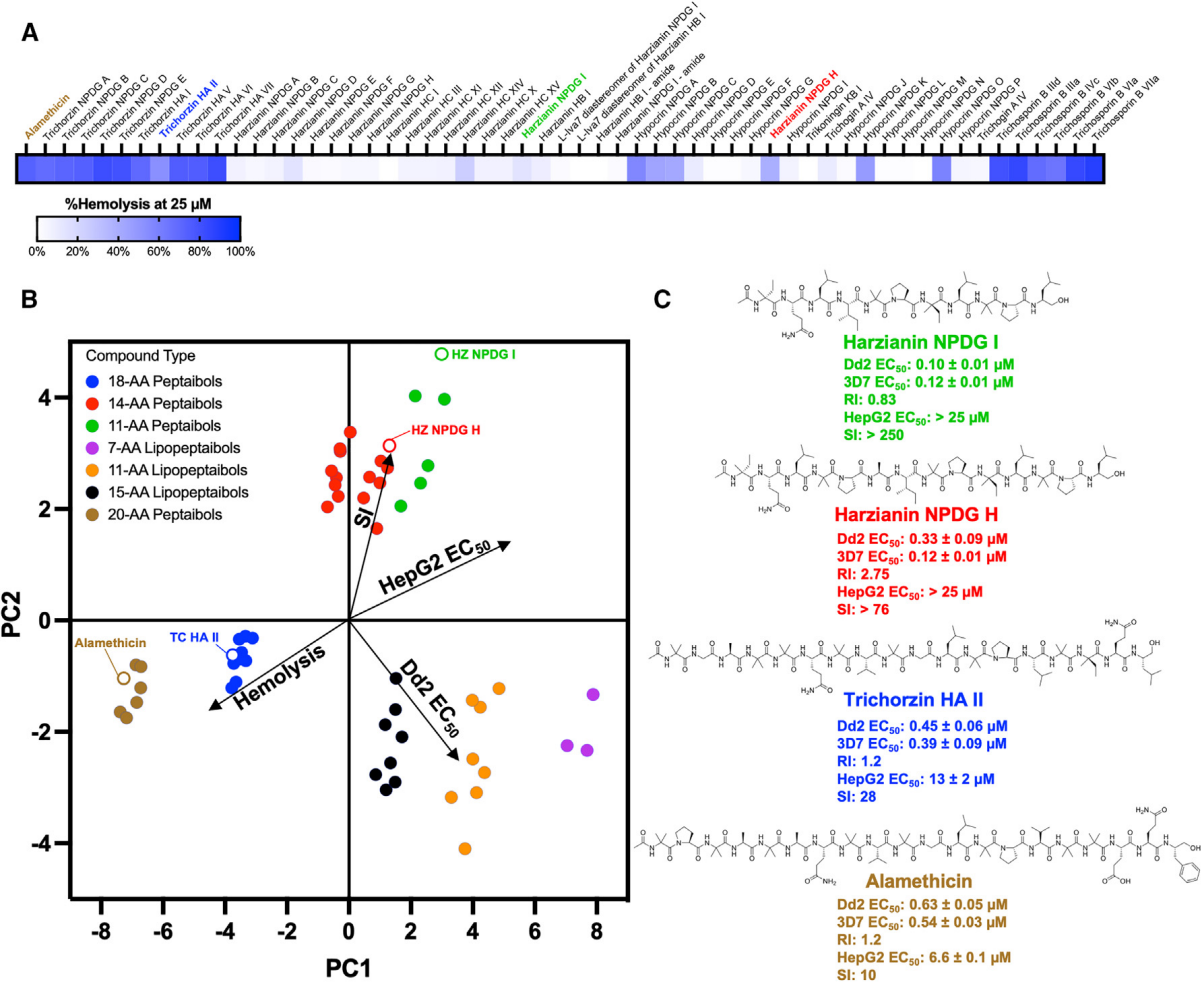
Peptaibol activity analysis (A) Hemolysis comparison of 57 peptaibols tested at 25 μM. (B) PCA highlighting relationships between inhibition, cytotoxicity, and hemolysis. Arrow length represents correlation strength. Arrow direction indicates positive/negative correlation. Peptaibols color-coded based on type. See also Figure S1. (C) Structures of *T. harzianum* peptaibols and alamethicin. Values represent mean ± SEM. Resistance index (RI) = Dd2 EC_50_/3D7 EC_50_, Selectivity index (SI) = HepG2 EC_50_/Dd2 EC_50_. Dd2 values for *T. harzianum* peptaibols originally communicated in PMID: 33565879. All testing performed in biological triplicate.

Following peptaibol incubation with RBCs, the release of hemoglobin was measured by spectrophotometry as an indication of hemolysis. Encouragingly, the majority of the peptaibols tested showed <50% hemolysis at the maximum concentration assessed (25 μM) (Figure 1A). To quantify which properties may be relevant to hemolysis, parasite activity, and cytotoxicity in HepG2, a principal component analysis (PCA) and Pearson correlation test were performed using assay calculated values for activity, as well as calculated and predicted chemical identifiers for all 57 peptaibols. These 27 PCA variables were chosen based on a comprehensive preliminary Pearson correlation matrix of 48 variables, as described in the STAR Methods section.

As seen in Figure 1B, peptaibols clustered primarily based on their length and lipidation status. Properties found to show no correlation, such as hemolysis and Dd2 EC_50_ show a ∼90° angle between loadings (indicated with arrows), and a ∼180° angle between inversely correlated variables such as hemolysis and HepG2 EC_50_ (Figure S1A). While antiplasmodial activity did not correlate with hemolysis, there was a strong correlation between peptaibol length and hemolysis (r = 0.6554, p < 0.0001), and a moderate inverse correlation between length and antiplasmodial activity (r = - 0.4199, p = 0.0011) (Figure S1B), suggesting shorter peptaibols were more potent and less hemolytic. There was also a strong inverse relationship between HepG2 EC_50_ and %hemolysis at 25 μM (r = - 0.7221, p < 0.0001), as predicted in the literature.^10^ While length was the primary determinant for hemolysis, other factors showed a strong influence on antiplasmodial activity. Those included the molecular complexity per atom (r = - 0.6877, p < 0.0001), the absence of a fatty acid group on the *N*-terminus (r = 0.6453, p < 0.0001), and the percentage of polar side chains (r = - 0.5867, p < 0.0001); more specifically, the percentage of proline residues (r = - 0.5052, p < 0.0001). We noted greater intergroup variability among the peptaibols and lipopeptaibols derived from *Hypocrea pachybasioides* (*H. pachybasioides*) compared to *Trichoderma harzianum* (*T. harzianum*) derived peptaibols. The compounds from *H. pachybasioides* also, on average, showed lower activity in Dd2, making them less desirable as antimalarial candidates.

### Peptaibols reduce AMA1/MSP1 in double-positive *P. berghei*

We chose to test a subset of these peptaibols against the *Plasmodium* liver stage in order to further explore these compounds’ therapeutic potential. To that end, a high content imaging (HCI) assay was performed to monitor any change in parasite luminescence, biomass, and inhibition of early and late maturation markers, MSP1 and AMA1, respectively. Peptaibols were tested post-sporozoite invasion at both 1 and 5 μM. Whereas luciferase inhibition was limited, several compounds reduced the percent of MSP1+ and AMA1+/MSP1+ parasites without impacting HepG2 (Figures S1C–S1E), suggesting a possible effect on parasite maturation. While compound potency was more modest than that seen in the blood stage, it does suggest the potential for liver stage inhibition for this compound class.

### Peptaibols chosen for further analysis indicate low cross resistance

Three *T. harzianum* peptaibols were selected for additional testing, including harzianin NPDG H (HZ NPDG-H), harzianin NPDG I (HZ NPDG-I), and trichorzin HA II (TC HA-II). Selection was based on parasite activity, cytotoxicity, and hemolytic potential. Each of the peptaibols chosen exhibited a selectivity index (SI) at least 1.8-fold higher than alamethicin—the most selective being the 11-AA peptaibol HZ NPDG-I with an SI > 250 and a Dd2 EC_50_ of 0.10 μM (Figure 1C). Structurally, the 18-AA TC HA-II shows a greater degree of similarity to known 20-AA inhibitor alamethicin, while the 14-AA HZ NPDG-H is more similar to HZ NPDG-I. Minor cytotoxicity was detected in TC HA-II and alamethicin, while no cytotoxicity was seen in HZ NPDG-H and HZ NPDG-I at the highest concentrations tested (25 μM). This toxicity may be due to cell lysis, as alamethicin and TC HA-II showed some hemolysis at 25 μM (73% and 53%, respectively). The initial activity screening was performed in Dd2,^35^ which is chloroquine (CQ) resistant due to a mutation in the DV transporter PfCRT.^36^ To examine any effects of this mutation, EC_50_ values in the CQ sensitive line 3D7 were also determined. No overt difference in activity was observed, with resistance indices (RIs) of 0.83 for HZ NPDG-I, 2.75 for HZ NPDG-H, and 1.2 for TC HA-II.

These 3 peptaibols were next probed for liver stage activity using HCI in *P. berghei*. Compounds were tested at 5 and 0.5 μM. Only TC HA-II showed luciferase inhibition at 48 HPI, potentially linked to its impact on HepG2 (Figure S1F). Interestingly, at 5 μM all 3 showed a decrease in the %AMA1+/MSP1+ parasites. Given this finding, we re-tested the most active peptaibol HZ NPDG-I in a dose-dependent luciferase assay and measured a *P. berghei* EC_50_ of 2.7 μM and without HepG2 toxicity up to 50 μM (Table S1).

HZ NPDG-I was tested in 3 additional *P. falciparum* lines (PfACS_A597, PfCARL_I1139K, and PfPI4K_S1320L) that contain mutations in PfACS,^37^ PfCARL,^38^ and PfPI4K,^39^ and a transgenic Dd2-ScDHODH line expressing the cytosolic type I DHODH from *S. cerevisiae* that can reveal compounds inhibiting mitochondrial DHODH.^40^ Compared to the parental lines,^41^ no substantial changes in inhibition were noted, with RIs ranging from 0.62 to 1.8 (Table S1). Parental line activity was found to be lower than previously recorded, with a Dd2 EC_50_ = 0.014 ± 0.006 μM, likely due to screening condition differences between the 2 laboratories, such as using synchronous culture and compound addition in early ring, a stage where peptaibols are more efficacious.

### Trichorzin HA II and harzianin NPDG I show a rapid killing profile

**Figure 2:**
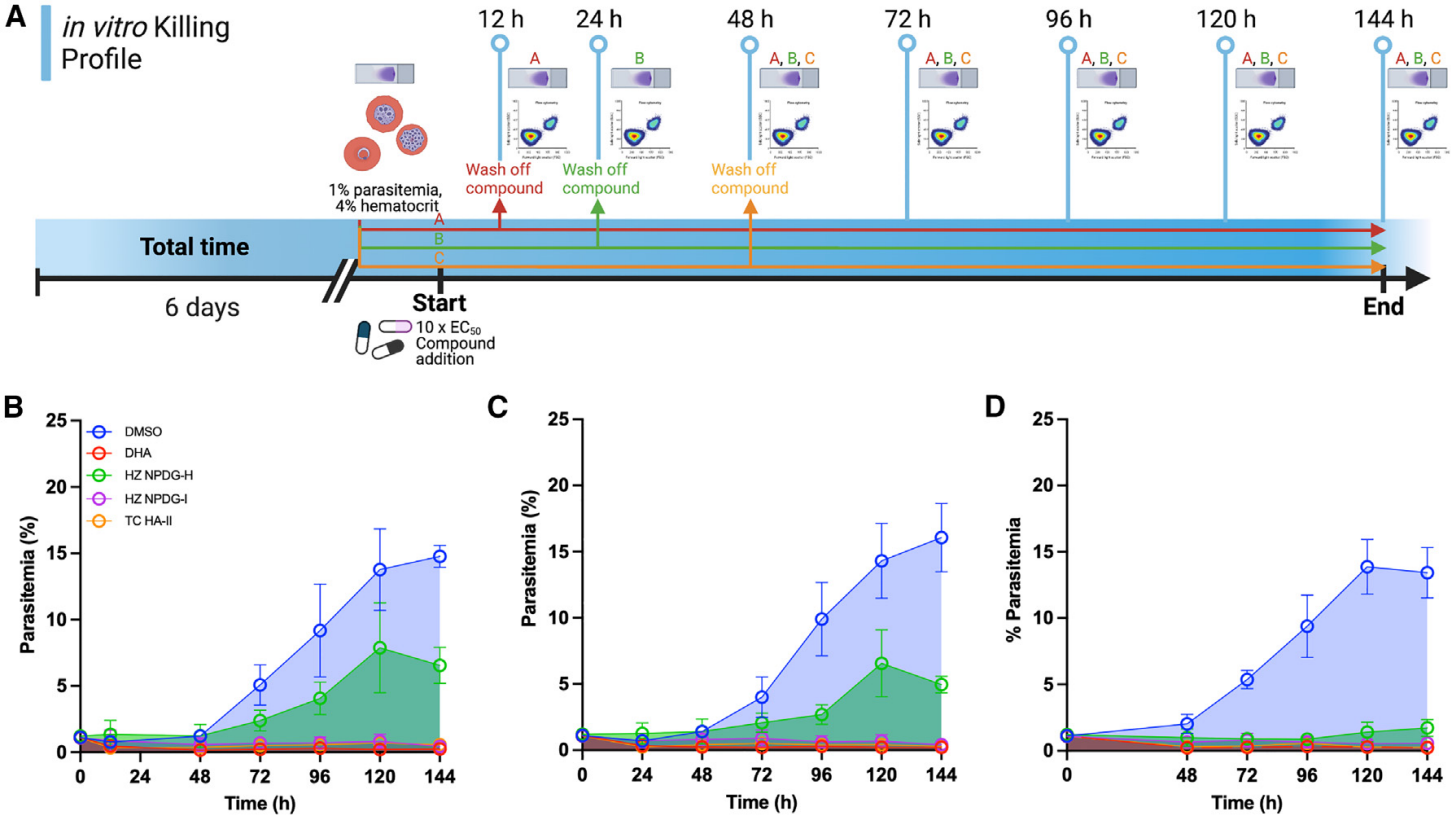
Peptaibol killing profile (A) Killing profile assay workflow. Dd2 parasites are exposed to a 10 x EC_50_ concentration of inhibitor prior to compound removal and monitoring with flow cytometry and Geimsa staining every 24 h. Parasitemia after 12 (B), 24 (C), or 48 h (D) incubation with peptaibols, vehicle, or DHA control. Data represent the mean ± SEM from 3 biological replicates. See Figure S2 for mitotracker staining.

We next examined the time-dependent killing profile of TC HA-II, HZ NPDG-H, and HZ NPDG-I. Asynchronous Dd2 cultures were incubated with a 10 x EC_50_ concentration of each compound or a vehicle control for 12, 24, or 48 h, prior to compound washout. Parasitemia and relative viability were monitored every day via flow cytometric staining with SYBR Green I and Mitotracker Deep Red FM (MTR) for a total of 6 days (Figure 2A). A 12 h incubation with HZ NPDG-I or TC HA-II was able to inhibit parasitemia for all 6 days; while HZ NPDG-H treated parasites recovered by day 3 (Figure 2B). In the first collection after 12 h incubation, HZ NPDG-H and HZ NPDG-I treated cultures showed an MTR signal similar to the vehicle control, followed by a sharp decrease at 48 h, suggesting a delayed effect on viability (Figure S2A). With HZ NPDG-I and TC HA-II, the fraction of non-viable parasites decreased over time but remained similar to the DHA control. After 24 h incubation, similar results were seen for all compounds, but HZ NPDG-H and HZ NPDG-I treated parasites showed no delayed effect on viability based on MTR staining (Figures 2C and S2B). In addition, the fraction of MTR^−^ parasites showed more consistency for all 6 days. No changes were seen in the 48 h treatment, except for HZ NPDG-H, which showed an increase in parasitemia at day 5 (Figure 2D). However, MTR staining with HZ NPDG-H remained comparable to the vehicle control, suggesting no impairment to viability and a cytostatic killing profile (Figure S2C).

### *T. harzianum* peptaibols demonstrate early stage inhibition

**Figure 3:**
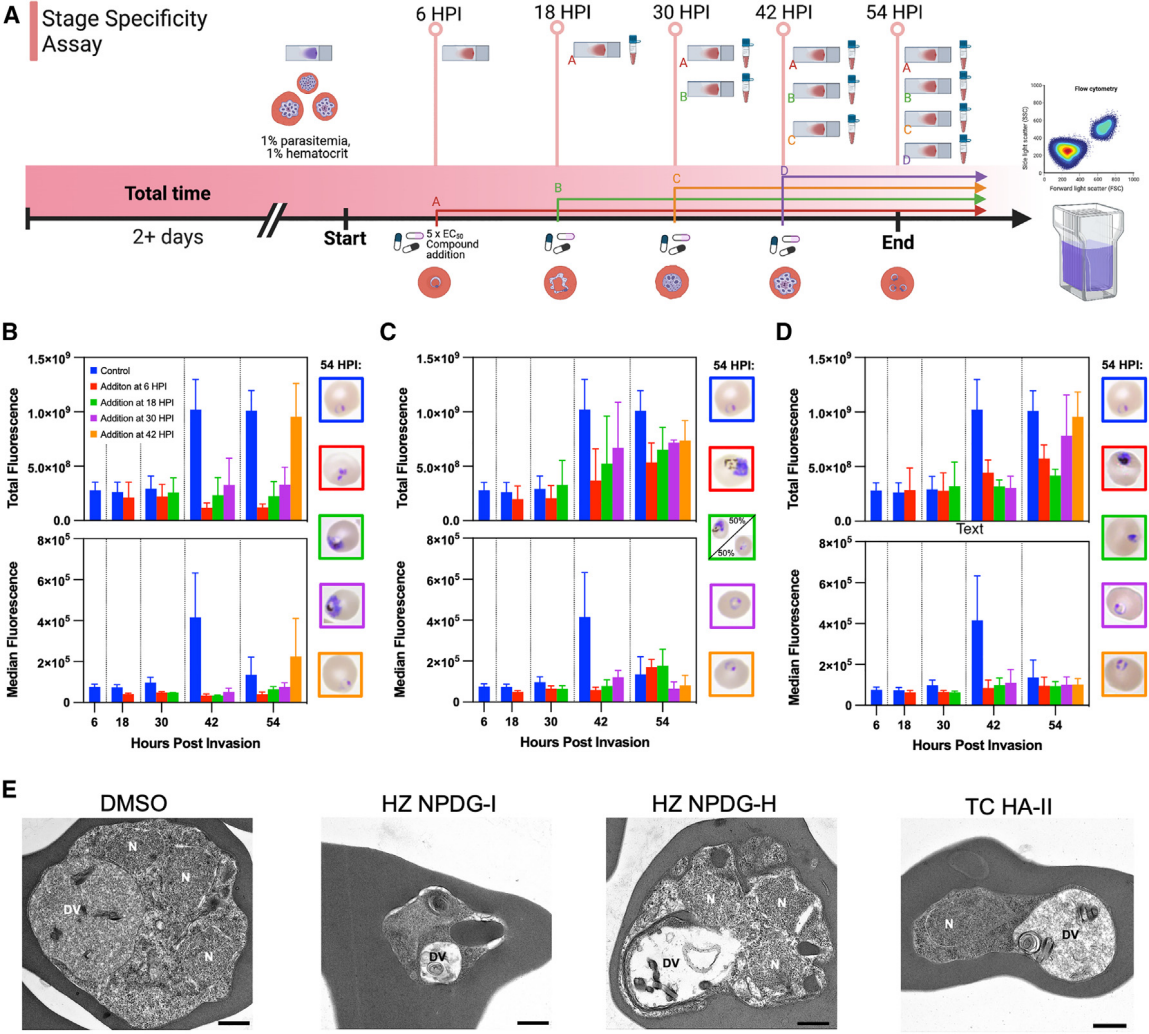
Peptaibol stage specificity (A) SSA workflow. After compound addition in ring-stage, samples are collected every 12 h for Giemsa staining and flow cytometry. (B) SSA results for HZ NPDG-I added at 5 x EC_50_. Different addition times represented by varying colors. Giemsa images shown from final collection (54 HPI). (C) SSA results for HZ NPDG-H added at 5 x EC_50_. (D) SSA results for TC HA-II added at 3 x EC_50_ (to avoid hemolysis). Data represent the mean ± SEM from 3 biological replicates. (E) TEM imaging of parasites 24 h after compound addition in ring. (N: nucleus, DV: digestive vacuole, scale: 500 nm). See also Figure S3.

To assess the stage specific activity of the selected peptaibols, we analyzed their inhibition phenotype at different stages of the intraerythrocytic life cycle. Dd2 parasites were synchronized and treated in either ring (∼6 HPI), trophozoite (∼18 HPI), late trophozoite/early schizont (∼30 HPI), or late schizont (∼42 HPI). Samples were collected every 12 h until control parasites reinvaded and new rings appeared (∼54 HPI) (Figure 3A). DNA content per parasite was determined with YOYO-1 staining and morphology with Giemsa staining of thin blood smears. The results indicated stronger inhibition for all peptaibols when added prior to late schizogony, however, each peptaibol showed distinctions. HZ NPDG-I, when added at 6 HPI, appears to arrest parasite development in early ring based on morphology and a fixed median fluorescence signal (Figures 3B and S3A). When added at 18 and 30 HPI, fluorescence inhibition is evident at all collection time points; yet, based on Geimsa staining, arrest occurs later in the trophozoite stage. Addition at 42 HPI showed no mean impact on total fluorescence, and parasites were able to reinvade and form rings.

With HZ NPDG-H, early exposure at 6 HPI was necessary for complete inhibition (Figures 3C and S3B). This corresponds to HZ NPDG-H showing a slower killing rate than its counterparts. Parasite arrest appears to occur in the late trophozoite to early schizont stage, with an increase in parasite size but no change in median fluorescence to indicate substantial multinucleation. Clearly identifiable DV abnormalities were also seen, such as content loss and fragmentation. HZ NPDG-H began to lose effectiveness when added at 18 and 30 HPI, with some replicates showing a dampened reinvasion peak at 42 HPI. A mixed population of abnormal rings and schizonts was seen with the 18 HPI addition and morphologically normal rings with the 30 HPI addition. Late-stage addition at 42 HPI showed only a small decrease in total fluorescence, with no noteworthy impairments.

Finally, TC HA-II showed a similar profile at both pre-schizont additions (6 and 18 HPI) with no sharp increase to total or median fluorescence and early arrest with a condensed permatroph morphology (Figures 3D and S3C). Addition at 30 HPI showed a delayed increase in total fluorescence at 54 HPI as opposed to 42. Despite this delay, parasites appeared normal on Giemsa smears with no obvious defects. As with HZ NPDG-H, addition at 54 HPI resulted in only a slight decrease in total fluorescence and no obvious change to morphology.

### Ultrastructural analysis shows DV abnormalities following peptaibol treatment

Several overt changes to the parasites digestive vacuole were noted during microscopic assessment of peptaibol treated culture, suggesting it may be a key site of action. The localization of these changes was consistent for each peptaibol; although disruptions were most obvious with HZ NPDG-H, possibly due to its slower killing rate resulting in later stage arrest and a larger DV. A loss of staining throughout the DV was the most common phenotype identified, with signs of detachment proximal to the DV. To better evaluate these changes, transmission electron microscopy (TEM) was performed on synchronous Dd2 parasites after compound exposure for 24 h in early ring. As seen in Figure 3E, a sharp contrast between the DV of control and peptaibol treated parasites can be seen, with a marked loss in vacuolar content and localized blebbing. HZ NPDG-H and NPDG-I showed similar malformations, including the appearance of abnormal vesicles and signs of perturbations to heme trafficking, such as an abundance of rounded hemoglobin (Figure S3D). Additionally, there was an apparent decrease in the number of neutral lipid bodies (NLB) identified in peptaibol treated parasites. The parasite plasma membrane (PPM) appeared generally intact following peptaibol addition, except for HZ NPDG-I which showed some patchy ribosomal loss around the outer edges of the parasite. No damage to the erythrocyte membrane was seen, consistent with our earlier findings that antiplasmodial activity is distinct from hemolysis. The size and stage of growth arrest by each peptaibol corresponded with the results of the stage-specific assay (SSA). Parasites treated with HZ NPDG-H showed some degree of multinucleation, while parasites treated with compounds HZ NPDG-I or TC HA-II were inhibited prior to schizogony.

### Peptaibols HZ NPDG-I and TC HA-II alter DV permeability

The detection of DV abnormalities following exposure to peptaibols prompted us to analyze changes in DV functions following peptaibol treatment. We first measured inhibition of β-hematin crystallization, a crucial step in the detoxification of heme in the DV. Despite the clear, dose-dependent inhibition seen from CQ control, no inhibition was seen with peptaibols at concentrations up to 200 μM (Figure S4A). Microscopic examination of treated wells also confirmed consistent crystal formation with all peptaibols, suggesting a mechanism distinct from CQ. Based on these findings, we redirected our focus to measuring DV pH and membrane integrity using FITC-dextran, a pH sensitive, membrane impermeable dye. After inoculating dye-loaded RBCs with parasites, cultures were maintained for a minimum of 96 h to allow dye accumulation in the DV. Fluorescence was then read in functionally isolated trophozoites following incubation with *T. harzianum* peptaibols, alamethicin, or V-type H^+^-ATPase inhibitor concanamycin A (CMA). The pH dependent fluorescence ratio (FI585/FI530) was then recorded after 0.5, 1, or 2 h. Rapid alkalization was seen after 0.5 h with TC HA-II and alamethicin (Figure S4A); while HZ NPDG-I showed a moderate increase in the florescence ratio after 1 h, followed by a significant (p = 0.0015) increase after 2 h. This slower, stepwise change more closely mirrored the ATPase pump inhibitor control CMA. No significant (p > 0.05) change was seen with the 14-AA peptaibol HZ NPDG-H. Microscopic analysis was then performed after 2 h to confirm the localization of membrane impermeable dye FITC-dextran. With TC HA-II, alamethicin, and HZ NPDG-I, most of the functionally isolated parasites no longer showed full colocalization of the dye within the DV, suggesting membrane leakage (Figure S4C). This effect was not seen with HZ NPDG-H or any of the controls, where fluorescence overlapped tightly with darkened hemozoin crystals.

### HZ NPDG-I induces channel activity in synthetic membranes

**Figure 4:**
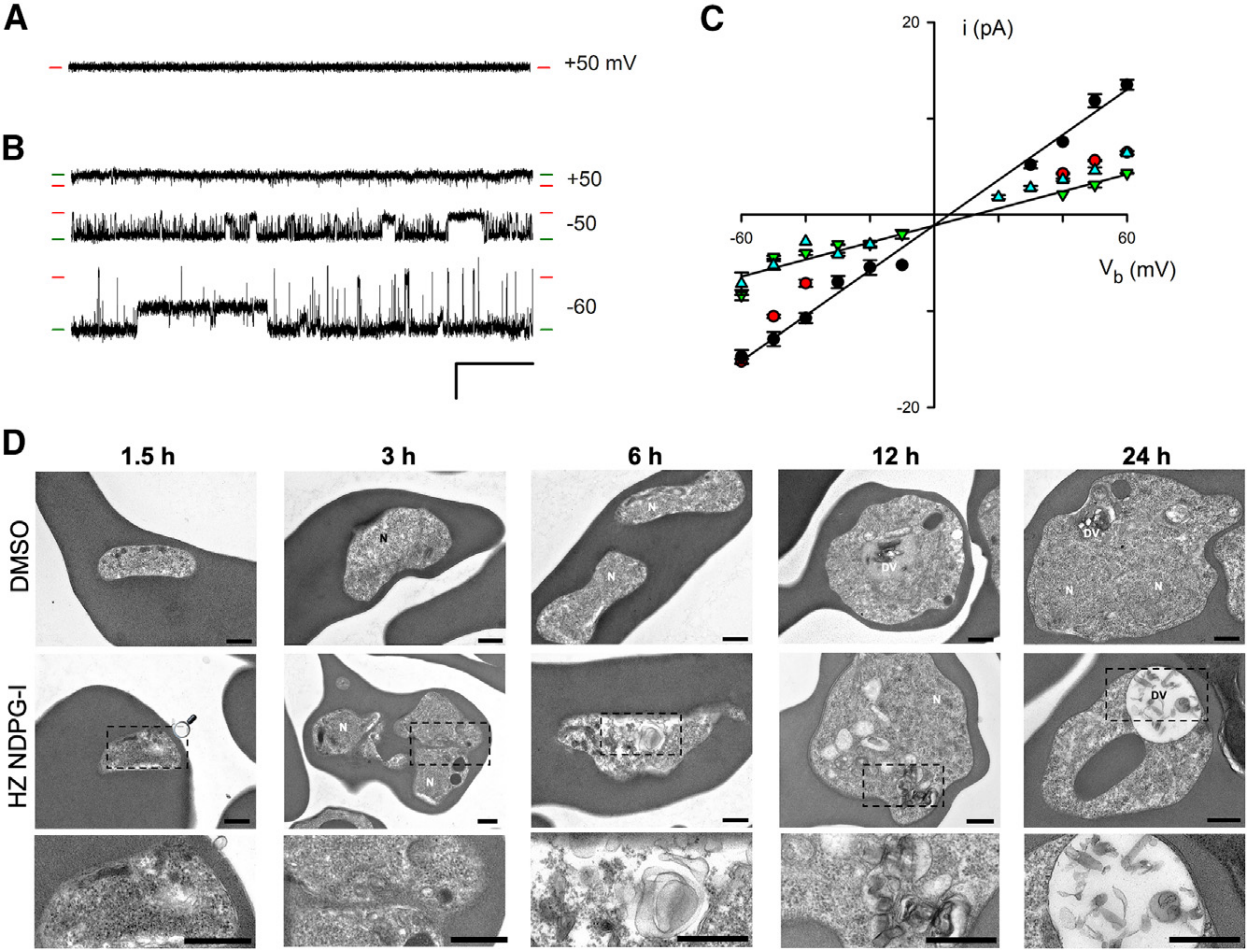
Electrophysiology and morphology with HZ NPDG-I (A) Current recording from planar lipid bilayer without peptaibol addition and reconstitution. Red dashes at ends of trace represent zero current level. Notice the stable baseline without channel-like transitions. (B) Recordings from separate bilayers after addition of 1 μM HZ NPDG-I. Red and green dashes represent closed and open channel levels, respectively. An intermediate current level is apparent in the bottom trace. Scale bar, 500 ms (horizontal)/10 pA (vertical). Imposed bilayer potentials (Vb) are indicated to the right of traces. (C) Current-voltage relationships for 4 separate channel molecules, with current amplitudes from individual channels represented with black circles, red circles, blue triangles, or green triangles. For each channel, symbols represent the mean ± S.E.M. current at indicated bilayer voltages (Vb). Solid lines reflect linear regression fits for the smallest and largest channels observed under our experimental conditions. (D) Timewise TEM of HZ NPDG-I treated parasites (N: nucleus, DV: digestive vacuole, scale: 500 nm).

Based on alamethicins’ functionality as a channel-forming peptide, we reasoned the *T. harzianum* peptaibols may be acting in a similar capacity in *Plasmodium*.^42^ To that end, we reconstituted these peptaibols into planar lipid bilayers and recorded induced currents to evaluate changes in membrane permeability. Lipid bilayers formed with 1,2-diphytanoyl-*sn*-glycero-3-phosphocholine (DPhPc) were stable and did not exhibit channel activity (Figure 4A). Addition of HZ NPDG-I to these bilayers reproducibly induced unambiguous channel-like activity (Figure 4B). These channels exhibited high open probabilities at most voltages. Interestingly, some recordings revealed sub-conductance states, apparent as transitions to levels intermediate to the channel open and closed states (Figure 4B, −60 mV trace). We tallied event amplitudes and found that channels produced by compound HZ NPDG-I incorporation exhibited ohmic responses to applied voltages, but that their conductances were variable (Figure 4C), with some incorporations producing greater ion flux through open channels. We analyzed 4 induced channels and found that their chord conductances ranged from 90 pS to 240 pS (4 separate symbols in Figure 4C), indicating high permeabilities. This variable conductance may reflect formation of channels that contain different numbers of peptaibol subunits, as reported with other channel-forming peptides.^43^ We did not attempt to determine the ion selectivity of HZ NPDG-I-induced channels; studies with derivatives of alamethicin, a model peptaibol, reveal weak selectivity that range from anion to cationselective depending on mutations in pore-lining residues.^44^ In contrast to HZ NPDG-I, addition of peptaibol HZ NPDG-H did not induce detectable channel activity (Figure S4C, showing 3 separate bilayers from 8 attempts). Preliminary studies with TC HA-II and Hypocrin NP B also did not reveal channel activity. Thus, HZ NPDG-I may increase permeability at 1 or more lipid bilayers in *P. falciparum*. Based on this, we reevaluated EC_50_ values after changing the *in vitro* lipid source for the parasites from Albumax II to human serum. A modest but consistent EC_50_ shift was seen with all peptaibols tested (Table S2), confirming a possible relationship between membrane lipid content and peptaibol activity. As seen with previous reports, control CQ showed greater inhibition in serum grown culture.^45^ While the EC_50_ values of HZ NPDG-H and I roughly doubled, the EC_50_ of 18-AA TC HA-II increased by nearly 5-fold. This could suggest better membrane permeability in Albumax II or a higher aggregation tendency for TC HA-II.

### HZ NPDG-I causes compounding changes to heme trafficking and compartmentalization

To better define the relationship between HZ NPDG-I and DV damage, we performed additional TEM imaging over the course of the parasite’s development, beginning in the ring stage. Some phenotypic variations in treated parasites were seen as early as 1.5 h post addition (Figure 4D). Minor signs of blebbing were noted and decreased cellular content. By 3 h, further signs of blebbing appeared along with abnormal membrane invaginations. There was a noted overabundance of rounded structures of undigested heme, suggesting some impairment in heme storage or transport. After 6 h, parasites that had formed a DV showed a marked loss in DV content. Impairments to heme compartmentalization continued to intensify after 12 h. At 24 h, the parasites showed further amplification of these changes, in addition to the appearance of membranous whirls throughout the cells.

### HZ NPDG-I causes transcriptional changes in vacuolar type proton transporters

**Figure 5:**
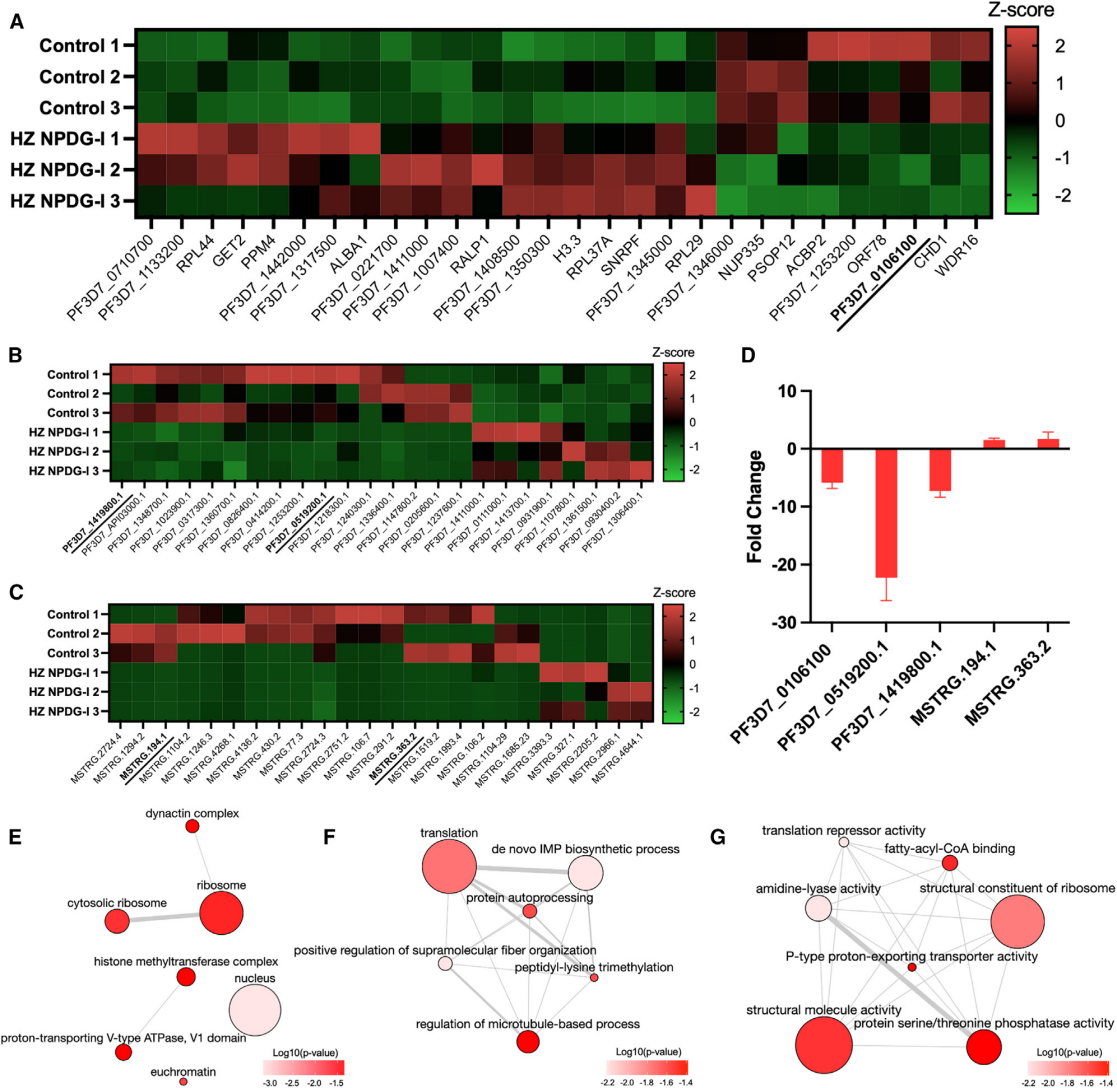
Transcriptome analysis of HZ NPDG-I (A) *Z* score value heatmap of mRNA (p < 0.05) identified with gene level sorting. RNA-seq performed on 3 biological replicates in 3D7 with HZ NPDG-I or vehicle control. Underlined transcripts validated via RT-qPCR. (B) Heatmap of mRNA identified with transcript level sorting (p < 0.05). (C) Heatmap of lncRNA (p < 0.05) identified. (D) RT-qPCR validation of selected transcripts from mRNA and lncRNA. Values represent the mean ± SEM from 3 biological replicates. (E) GO analysis of significant (p < 0.05) mRNA for cellular component terms. Node color represents the log10(p value), node size represents the number of annotations per term in the EBI. (F) GO analysis of significant (p < 0.05) mRNA for biological process terms. (G) GO analysis of significant (p < 0.05) mRNA for molecular function terms.

To better understand the effect of HZ NPDG-I on global transcription, we performed an RNA-seq analysis on 3 biological replicates of synchronous 3D7 culture exposed for 1 h to an EC_50_ concentration of peptaibol or vehicle control. Differential expression changes were identified in both messenger RNA (mRNA) and novel long noncoding RNA (lncRNA) (Figures 5A–5C, Table S3). For mRNA, differential expression was analyzed with both gene level sorting (all isoforms grouped as one) and transcript level sorting (each isoform weighed separately) for increased fidelity. For lncRNA, *cis*-target prediction analysis was also performed. We noted that several transcripts for transporters of the ER/Golgi and DV membranes were identified as being differentially expressed, including V-type proton ATPase subunit C (PF3D7_0106100) with gene sorting results, and V-type proton ATPase 16 kDa proteolipid subunit (PF3D7_0519200) with transcript sorting results, lncRNA MSTRG.194.1 with the predicted *cis*-target Sec61-gamma (PF3D7_0210000) – a glutathione transporter component, and MSTRG.363.2 with the predicted *cis*-target ER membrane protein complex subunit 5 (EMC5, PF3D7_306700)—an Mg^2+^ transporter. Several key enzymes were also dysregulated, including glutathione reductase (PF37D_1419800, transcript level), *cis*-prenyltransferase (PF3D7_0826400, transcript level), and adenylosuccinate lyase (PF3D7_0206700, gene level).

To validate these results, 5 transcripts with possible compound relevance were assessed using RT-qPCR. These included, mRNA transcripts PF3D7_0106100 (RNA-seq log2fc: −1.35), PF3D7_0519200.1 (RNA-seq log2fc: −5.06), PF37D_1419800.1 (RNA-seq log2fc: −3.23), and lncRNA MSTRG.194.1 (RNA-seq log2fc: 16.51) and MSTRG.363.2 (RNA-seq log2fc: 14.54). The resulting CT values were normalized to housekeeping gene serine-tRNA ligase (PF3D7_0717700). Results obtained with RT-qPCR (Figure 5D) showed a similar regulation pattern to the RNA-seq analysis, with a mean fold change of −5.86 for F3D7_0106100, −22.26 for PF3D7_0519200, −7.27 for PF37D_1419800, 1.52 for MSTRG.194.1, and 2.56 for MSTRG.363.2; confirming the dysregulation of membrane transport components in response to HZ NPDG-I incubation.

To better understand the potential impact of the observed dysregulation, GO enrichment analysis was performed on the differentially expressed (DE) mRNA identified from the gene-level sorting. As seen in Figure 5E, cellular component GO-terms were enriched for nucleus, euchromatin, histone methyltransferase complex, proton-transporting V-Type ATPase, V1 domain, and ribosome-related terms. The terms enriched for biological process included supramolecular fiber organization, microtubule related terms, and terms related to protein translation and processing (Figure 5F). Finally, the GO-terms most notably enriched in regards to molecular functions were P-type proton-exporting transporter activity and fatty acyl-CoA binding (Figure 5G). Taken together, many of these terms relate to proton-transport at the DV and protein processing, both of which may implicate the DV as the site of action.

### Resistance line generation identifies mutations in DV localized transporter PfMDR1

**Figure 6:**
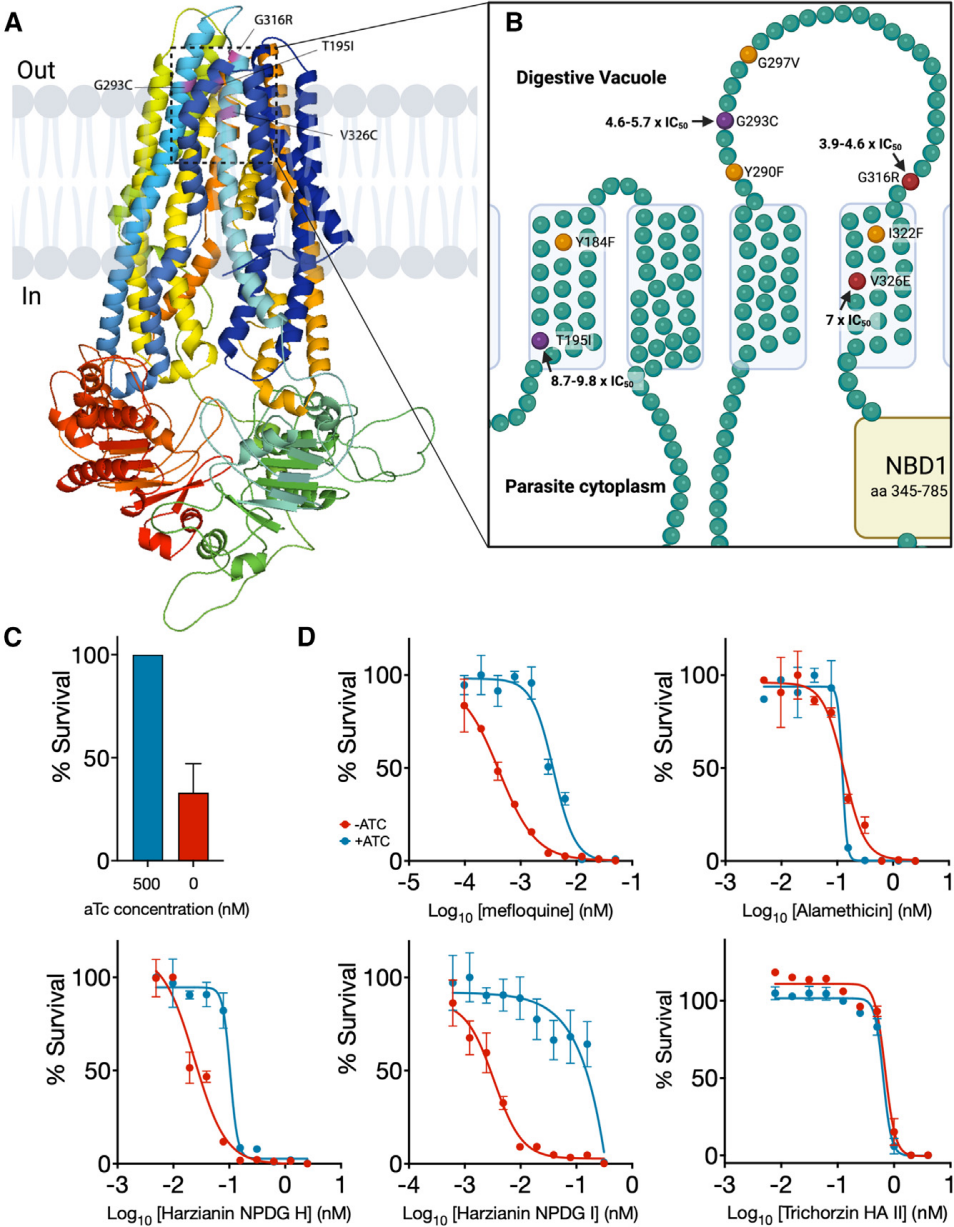
Relationship of *Pf*MDR1 and select peptaibols (A) Homology model for *Pf*MDR1 created using Swiss-Model and PDB structure, 7a69.1. Nanodisc reconstituted human ABCB1 in complex with MRK16 Fab and vincristine.^47^ (B) Simplified mutation map for *Pf*MDR1. Mutations identified in HZ NPDG-I-resistant clones shown in purple, previously reported proximal mutations for *Pf*MDR1 shown in yellow. Overlapping mutations shown in red.^46^ (C) Percent parasite survival upon aTc removal in PfMDR1 cKD. (D) Dose-response curves in *Pf*MDR1 cKD for peptaibols and control mefloquine. Knockdown with ATC in blue, without ATC in red. Values represent the mean and SEM of three biological replicates.

To investigate the possible mechanism of action and resistance to HZ NPDG-I, Dd2 parasites were *in vitro* evolved to resist the compound through gradual ramp-up in exposure. Nine clonal parasite lines from 3 independent selections were isolated, each exhibiting EC_50_ values 4 to 10-fold higher than the parental line. These clones were then subjected to whole genome analysis (Table S4). Compared to the drug sensitive parent, the mutant clones had acquired only a few fully penetrant nonsynonymous or disruptive changes. These mutations were in the glutamate dehydrogenase gene GDH3 (PF3D7_0802000), ApiAP2G (PF3D7_1222600)—a transcription factor linked to parasite sexual development that is frequently mutated during experiments that involve long term culture, and in PF3D7_0927600, coding for a predicted RNA binding protein. As the Dd2 strain contains several copies of the pfmdr1 locus, we next searched for heterozygous changes. Strikingly, all HZ NPDG-I-resistant clones acquired nonsynonymous mutations in *Plasmodium* multidrug resistance 1 transporter (pfmdr1) (PF3D7_0523000), with all clones containing 1 or 2 “wild type” and 1 or 2 mutant copies of pfmdr1. *Pf*MDR1 is an ABC transporter commonly associated with the DV that has been previously implicated in drug resistance to CQ, amodiaquine, and others.^46^^,^^47^ We constructed a pfmdr1 homology model using a cryoEM structure of the human ABCB1 transporter, which showed that all 4 of these mutations were located in the channel region near the predicted binding site of vincristine (Figure 6A). Of the SNPs we identified, T195I and V326C were associated with a higher level of resistance to HZ NPDG-I (8 to 10-fold), while G293C and G316R seemed to impart a lower degree of resistance (3 to 6-fold) (Figure 6B). For inhibitors such as CQ, mutations in this transporter are suspected to reduce import of the drug to the DV.^48^ Future studies exploring the role of these *Pf*MDR1 mutations are planned using labeled peptaibols to measure the impact on cellular trafficking.

### *Pf*MDR1 protein knockdown induces differential sensitivity to HZ NPDG H and I

The essentiality of *Pf*MDR1 in intraerythrocytic parasites was determined by maintaining cells in the presence and absence of aTc, which controls expression of the target transcript, then analyzing parasite growth using luminescence. Results revealed that parasites maintained in aTc were able to progress through the life cycle, while aTc withdrawal resulted in growth perturbation, implicating the essentiality of the PfMDR1 protein (Figure 6C). *Pf*MDR1 has been shown to transport structurally diverse antimalarial drugs,^49^ and this activity is modified when mutations are introduced to the protein, resulting in altered drug susceptibility of mutant lines in culture.^50^^,^^51^ We screened our conditional knockdown (cKD) line against mefloquine—one of the drugs that *Pf*MDR1 has been shown to transport—and observed a ∼12-fold shift in sensitivity when the protein was knocked down (IC_50_ = 5 nM), compared to when testing in the wild-type YFP-expressing NF54 (IC_50_ = 0.4 nM) (Figure 6D). *Pf*MDR1 knockdown hypersensitized parasites to the peptaibol HZ NPDG-I, with a 43-fold shift in activity. Additionally, a lesser fold change in sensitivity to HZ NPDG-H (∼6-fold) was seen. However, no differential sensitivity to TC HA-II and alamethicin was observed. Differential sensitivity was not observed for all tested compounds with or without aTC in the control line.

Given the differences in sensitivity of this subset of peptaibols to PfMDR1 cKD, we returned to test their activity in the clones previously generated through the *in vitro* evolution of resistance to HZ NPDG-I. Alamethicin, TC HA-II, and HZ NPDG-H were screened in 4 of the resistant clones, each containing 1 of the 4 PfMDR1 mutations identified. None of the peptaibols tested showed a similar magnitude shift in activity from the parental line compared to HZ NPDG-I (Figure S5); although HZ NPDG-H did show a minor 1.4-fold EC_50_ increase. Combined with the sensitivity in the cKD, a link between PfMDR1 and HZ NPDG-H activity is possible, abet to a lesser degree than HZ NPDG-I.

## Discussion

The results of our study provide foundational knowledge on the antiplasmodial activity of peptaibols, a unique compound class not previously expanded upon in the malaria parasite. These and other AMPs have demonstrated broad activity in drug resistant bacteria and cancer cells, linked to their abilities to (1) disrupt membranes, and (2) translocate to the cytoplasm and interact with various targets.^52^ Unlike cationic AMPs, peptaibols contain few charged or polar residues, making them less dependent on cell surface charge for activity.^19^ The broad effects of these mechanisms are advantageous compared to single target antibiotics due to the higher evolutionary hurdle of drug resistance.

During our initial multi-compound comparison, we found that peptaibol antiplasmodial potency was correlated with shorter AA length, complexity per atom, and the number of polar AA residues. Traditionally, the peptaibols explored for antimicrobial activity have been longer analogs such as alamethicin.^22^^,^^33^ More recently, shorter peptaibols with pronounced bioactivity have been identified, despite their inability to span bilayers as monomers.^19^ Our findings indicate that longer peptaibols are, on average, more hemolytic and cytotoxic. We analyzed the impact of peptaibol lipidation status, and found lipopeptaibols showed lower antiplasmodial activity, and poor selectivity. Studies with the lipopeptaibol trichogin GA IV suggest it acts through a “carpet-like” mechanism of action, with the helical peptide portion in plane with the bilayer and the fatty acyl chain oriented toward the polar membrane face.^53^^,^^54^^,^^55^ Additionally, lipopeptaibols were shown to be less affected by membrane cholesterol content compared to nonlipidated peptaibols, resulting in low specificity.

With TEM, we found that our most active peptaibols showed some phenotypic similarities, including DV fragmentation, a loss of DV content, the formation of abnormal vesicles, and a dramatic loss of NLBs. While NLBs are often proximally linked to the DV, their functional connection remains enigmatic.^48^ Further microscopy with fluorescently tagged peptaibols could shed light on this and other connections. It is possible DV impairment appears most prominent due to its role as a site of drug accumulation, allowing the DV to reach an effective peptaibol concentration prior to other organelles.^56^ Research in the protozoan parasites *Leishmania infantum* found that peptaibol inhibition was linked to impairment in mitochondrial membranes.^28^ While we noted no obvious impact to the *Plasmodium* mitochondrion, activity in this organelle might explain inhibition in the *Plasmodium* liver stage where a DV is absent.

Further analysis of the most active analog, HZ NPDG-I, showed that it was the only test peptaibol to induce channel-like activity in synthetic phosphocholine membranes, despite being too short to span a bilayer. As a result, HZ NPDG-I likely acts either by bending the membrane around it in a “toroidal-pore” like mechanism or by doubling up end to end to form a “barrel-stave” channel.^18^ The resulting impact on the parasite occurs rapidly. While we were unable to identify a single molecular target through the *in vitro* evolution of resistance, we did show inhibition was reduced through mutations in DV transporter *Pf*MDR1. These mutations may affect the rate of accumulation of HZ NPDG-I in the DV and possibly elsewhere, increasing the EC_50_ as a result. Interestingly, cKD of *Pf*MDR1 resulted in increased sensitivity to both HZ NPDG-I and HZ NPDG-H but not the longer peptaibols TC HA-II and alamethicin. Sensitivity to the 11-AA HZ NPDG-I was several orders of magnitude greater than that seen for the 14-AA HZ NDPG-H. These results again point toward peptaibol length as a key governing factor for *in vitro* activity.

**Figure 7:**
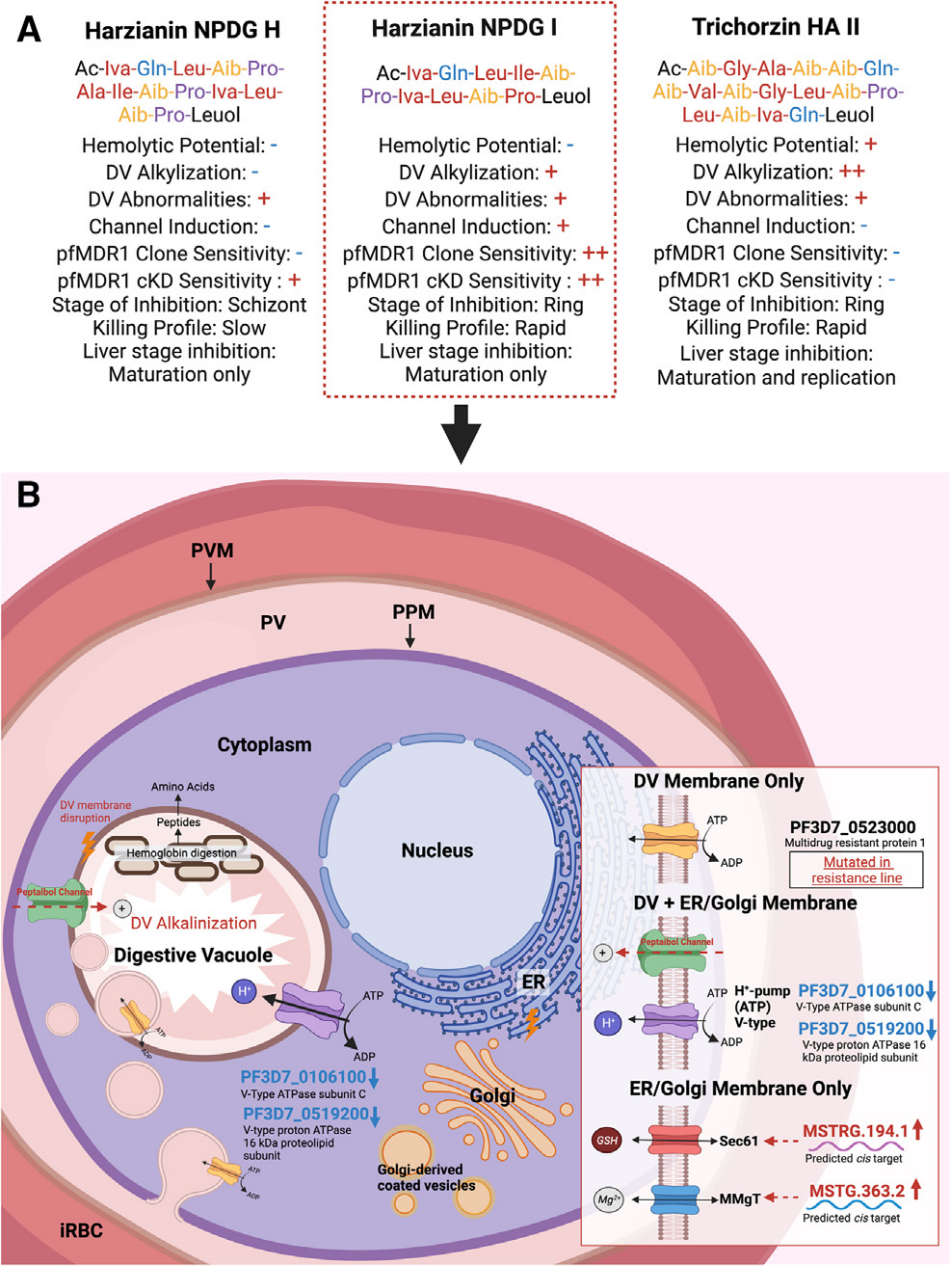
Peptaibol activity summary (A) Combined findings for the peptaibols explored in this study. A “–” indicates a negative finding, a single “+” indicates a positive finding, and a double “++” indicates a strong positive finding. (B) Proposed mechanism of action for HZ NPDG-I.

Analysis of these peptaibols underscores the wide range of activity seen among this understudied compound class and implicates *Plasmodium* specific trends (Figure 7A). An extended analysis of HZ NPDG-I suggested its site of action is the parasites DV membrane, supported by induced DV abnormalities including alkalinization and membrane disruption and the transcriptional dysregulation of DV membrane transporters (Figure 7B). This finding was further supported by the evolution of resistance to HZ NDPG-I through a PfMDR1 mutation and its sensitivity in a PfMDR1 cKD line. Given the identified potential of this compound to induce channel activity in DV-like membranes, HZ NPDG-I could act through ion channel formation. Considering that PfMDR1 transport seems essential for HZ NPDG-I but less so for HZ NPDG-H and TC HA-II which did not induce channel activity, they may act through an independent mechanism, or rely on a different primary site of action.

### Limitations of the study

While this study represents the first of its kind to explore the antiplasmodial activity of peptaibols, it has limitations. First, our findings are primarily based on *in vitro* experiments and may not fully reflect *in vivo* conditions. Optimization of oral bioavailability of 11 AA peptaibols is needed for *in vivo* efficacy. Second, the exact mechanisms of action of peptaibols remain unclear, as we were unable to clearly define a molecular target. Additionally, while our study included 57 peptaibols, expanding to additional peptaibols could enhance the validity and generalization of our findings. Further research, including *in vivo* studies and safety assessments, is necessary to validate the potential of peptaibols as antimalarial agents.

## Significance

**There is a need for novel antimalarials to combat the development of drug resistant *Plasmodium* parasites. In this study, we assess the potential of peptaibols, a compound class being utilized to combat drug resistance in other organisms. Unlike single target drugs, peptaibols may interact with several intracellular targets, reducing the likelihood evolved resistance. While off-target effects are often a concern for multitarget inhibitors, our data suggest a high potential for selectivity. Additionally, we identified variables that may contribute to hemolysis, a major factor in peptide toxicity. Our analysis of a peptaibol subset demonstrated their *in vitro* activity varies in relation to the killing rate and stage dependence. Despite this, all 3 displayed a significant morphological and functional impact on the parasite’s DV. HZ NPDG-I, showed channel activity in synthetic phosphocholine membranes, suggesting the ability to form ion channels on DV-like membranes. These findings align with the identification of a PfMDR1 mutation in HZ NPDG-I-resistant lines, and the compounds sensitivity to PfMDR1 knockdown. Together, these results offer insight into the *Plasmodium* specific activity of peptaibols.**

## STAR★Methods

### Key resources table

**Table:** 

REAGENT or RESOURCE	SOURCE	IDENTIFIER
**Antibodies**		

Rat anti-AMA1	BEI Resources	MRA-897
Rabbit anti-*P. berghei* MSP1	Hanson Lab, UTSA	N/A

**Chemicals, peptides, and recombinant proteins**		

Harzianin NPDG I	Cichewicz lab, University of Oklahoma; OU, USA	Ac**-**D-Iva-L-Gln-L-Leu-L-Ile-Aib-L-Pro-D-Iva-L-Leu-Aib-L-Pro-L-Leuol
Harzianin NPDG H	Cichewicz lab, University of Oklahoma; OU, USA	Ac-D-Iva-L-Gln-L-Leu-Aib-L-Pro-_L-_Ala-L-Ile-Aib-L-Pro-D-Iva-L-Leu-Aib-L-Pro-L-Leuol
Trichorzin HA II	Cichewicz lab, University of Oklahoma; OU, USA	Ac-Aib-Gly-L-Ala-Aib-Aib-L-Gln-Aib-L-Val-Aib-Gly-L-Leu-Aib-L-Pro-L-Leu-Aib-D-Iva-L-Gln-L-Leuol
L-Iva diastereomer of harzianin NPDG I	Cichewicz lab, University of Oklahoma; OU, USA	Ac**-**D-Iva-L-Gln-L-Leu-L-Ile-Aib-L-Pro-L-Iva-L-Leu-Aib-L-Pro-L-Leuol
L-Iva diastereomer of harzianin HB I	Cichewicz lab, University of Oklahoma; OU, USA	Ac**-**Aib-L-Asn-L-Leu-L-Ile-Aib-L-Pro-L-Iva-L-Leu-Aib-L-Pro-L-Leuol
Harzianin HB I – amide	Cichewicz lab, University of Oklahoma; OU, USA	Ac**-**Aib-L-Asn-L-Leu-L-Ile-Aib-L-Pro-D-Iva-L-Leu-Aib-L-Pro-L-Leu-NH_2_
Harzianin NPDG I – amide	Cichewicz lab, University of Oklahoma; OU, USA	Ac**-**D-Iva-L-Gln-L-Leu-L-Ile-Aib-L-Pro-D-Iva-L-Leu-Aib-L-Pro-L-Leu-NH_2_
Alamethicin	Abcam	Cat#ab141893; Ac-Aib-Pro-Aib-Ala-Aib-Ala-Gln-Aib-Val-Aib-Gly-Leu-Aib-Pro-Val-Aib-Aib-Glu-Gln-Phl
SYBR Green I	Invitrogen	Cat#S7567
MTS reagent	Promega	Cat#PR-G3581
FITC-dextran	TCI America	Cat#F0918100MG
L-a-Phosphatidylcholine	Sigma-Aldrich	Cat#P3556-100MG
Hemin	Sigma-Aldrich	Cat#H9039-1G
YOYO-1	Invitrogen	Cat#Y3601
Paraformaldehyde	Sigma-Aldrich	Cat#P6148-500G
Anhydrotetracycline	Sigma-Aldrich	Cat#37919
Blasticidin S	RPI Corp	Cat#B12150–-0.1
2% Paraformaldehyde/2.5% Glutaraldehyde in 0.1M Sodium Cacodylate buffer	Electron Microscopy Sciences	Cat#15960-01
Cacodylate buffer	Electron Microscopy Sciences	Cat#11652
Mitotracker Deep Red FM	Invitrogen	Cat#M22426

**Critical commercial assays**		

Direct-zol RNA MiniPrepPlus	Zymo Research	Cat#R2070
SuperScript First-Strand Synthesis System	Invitrogen	Cat#11904-018
SYBR Select Master Mix	Life Technologies	Cat#4472903
Renilla-Glo(R) Luciferase Assay System	Promega	Cat#E2750

**Deposited data**		

Harzianin NPDG I-selected resistance line sequencing	NCBI Sequence Read Archive	Accession #PRJNA812929
Harzianin NPDG I-RNASeq Data	NCBI Sequence Read Archive	Accession #PRJNA1018932

**Experimental models: Cell lines**		

Parasite line 3D7	BEI Resources, MRA-102	N/A
Parasite line Dd2 (Chakrabarti Lab)	BEI Resources, MRA-150	N/A
Parasite line Dd2 B2 (Winzeler lab)	PMID: 29326268	N/A
Parasite line PfACS_A597	PMID: 34348113 gifted via the David Fidock lab	N/A
Parasite line PfCARL_I1339K	PMID: 27381290	N/A
Dd2-ScDHODH	Transgenic parasite line expressing Saccharomyces cerevisiae dihydroorotate dehydrogenase, PMID: 17330044	N/A
Parasite line PfPI4K_S1320L	PMID: 24284631	N/A
PbLuc, Parasite line P. berghei-ANKA-GFP-Luc-SMCON	Insectary Core Facility, New York University	N/A
PfMDR1 cKD Parasite line	Niles Lab	N/A
Parasite Line NF54	Niles Lab	N/A
Human hepatocyte line HepG2 (Chakrabarti Lab)	ATCC HB-8065	N/A
Human hepatocyte line HepG2 (Winzeler lab)	ATCC HB-8065	N/A
Human hepatoma HepG2 (Hanson lab)	STR profiled by ATCC to confirm identity (barcode STRA-1292)	N/A
Parasite line *P. berghei* ANKA 676m1cl1 (Hanson lab)	BEI Resources (MRA-868) for the UGA Sporocore	*P. berghei* ANKA

**Experimental models: Organisms/strains**		

*P. falciparum* asexual blood stage parasites	BEI Resources, MRA-102	N/A
*P. falciparum* asexual blood stage parasites	BEI Resources, MRA-150	N/A
*P. falciparum* asexual blood stage parasites	Winzeler Lab	N/A
*P. falciparum* asexual blood stage parasites	Niles Lab	N/A
*P. berghei* liver stage parasites	Winzeler Lab	N/A
*H. sapiens* hepatocellular carcinoma cells	ATCC HB-8065	N/A
*H. sapiens* hepatocellular carcinoma cells	Winzeler Lab, HepG2-A16-CD81	N/A

**Oligonucleotides**		

**RT-qPCR primers**	Listed in Table S5.	N/A
PfMDR1 RHR forward sequence	gtacggtacaaacccggaattcgagctcggGCAT TTATATGATCCGCAAA	N/A
PfMDR1 RHR reverse sequence	aagacgagagattgggtattagacctagggat aacagggtaatCCCATAT ATACTATACATATATATTATCATTTAG	N/A
PfMDR1 sgRNA target site	GGAACCTTTGTACAGTCACA	N/A

**Recombinant DNA**		

pSN054	CRISPR donor vector, PMID: 33431920	N/A
LHR and re-codonized region (3736–4257 bp; stop codon removed)	ATGTTGGACCATATGGTAAAAGCTTA TCAGGTGGA CAAAAACAGAGAATAGCTATAGCT AGAGCATTATT AAGAGAACCTAAAATATTATTATTA GATGAAGCAA CATCATCACTTGATTCCAATTCTG AGAAATTAATT GAAAAAACTATTGTAGATATTAAA GATAAAGCTGA CAAAACTATTATTACTATTGCCCA CAGAATTGCAT CTATAAAACGATCAGACAAAATTG TGGTATTTAAT AACCCTGACAGAAATGGAACCTTC GTCCAGAGCCA CGGCACGCACGATGAATTGTTGTC TGCTCAGGACG GGATCTATAAGAAGTATGTGAAAC TGGCTAAA	N/A

**Software and algorithms**		

GraphPad Prism Version 9	GraphPad Software; San Diego, CA, USA	www.graphpad.com
Cytoscape Version 3.91	Institute for Systems Biology; Washington, USA	http://cytoscape.org
FlowJo Version 10	Becton, Dickinson and Company; Ashland, OR, USA	www.flowjo.com
Biorender	Biorender; Toronto, Canada	https://biorender.com
CDD Vault	Collaborative Drug Discovery, Inc, Burlingham, CA, USA	https://www.collaborativedrug.com
Cutadapt	https://doi.org/10.14806/ej.17.1.200	https://cutadapt.readthedocs.io/en/stable/
FastQC	Andrews, S., FastQC: A Quality Control Tool for High Throughput Sequence Data. 2010.	https://www.bioinformatics.babraham.ac.uk/projects/fastqc/
Bowtie2	Langmead B, Salzberg S. Fast gapped-read alignment with Bowtie 2. Nature Methods. 2012, 9:357-359.	https://bowtie-bio.sourceforge.net/bowtie2/index.shtml
HISAT2	https://doi.org/10.1101/gr.275193.120	http://daehwankimlab.github.io/hisat2/
StringTie	https://doi.org/10.1038/nbt.3122	https://ccb.jhu.edu/software/stringtie/#install
Gffcompare	https://doi.org/10.12688/f1000research.23297.1	https://ccb.jhu.edu/software/stringtie/gffcompare.shtml
edgeR	https://doi.org/10.1093/bioinformatics/btp616.	https://bioconductor.org/packages/release/bioc/html/edgeR.html
Instant Clue	https://doi.org/10.1038/s41598-018-31154-6	http://www.instantclue.uni-koeln.de/
Revigo	https://doi.org/10.1371/journal.pone.0021800	http://revigo.irb.hr/
PlasmoDB	The Plasmodium Genome Consortium (2001) PlasmoDB: an integrative database of the Plasmodium falciparum genome.	https://plasmodb.org/plasmo/app

### Resource availability

#### Lead contact

Information and requests for resources should be directed to Debopam Chakrabarti, (dchak@ucf.edu).

#### Materials availability

Chemical structures and ^1^H and ^13^C NMR for the 52 *T harzianum* and *H. pachybasioides* peptaibols published previously in PMID: 33565879. Spectra for trichorzin HA II and the 4 new 11-AA peptaibols not previously published, included in Data S1. UPLC-MS spectra for the main peptaibols used in this study and purity information for alamethicin also included in Data S1, along with peptide sequences.

#### Data and code availability

• Sequencing data for HZ-NPDG I-selected parasite samples NCBI SRA: PRJNA812929 and RNASeq data for control and HZ-NPDG I NCBI SRA: PRJNA1018932 have been deposited at the NCBI Sequence Read Archive and are publicly available as of the date of publication. Accession numbers are listed in the key resources table. Microscopy data reported in this paper is available upon request.
• This paper does not report original code.
• Any additional information required to reanalyze the data reported in this paper is available from the lead contact upon request.

### Experimental model and subject details

Asexual blood stage parasites grown in the Chakrabarti laboratory were cultured based on a protocol by Trager and Jensen^57^ with some modifications.^35^ Culture was maintained at 4% hematocrit with human A+ whole blood obtained from the Florida OneBlood, in RPMI 1640 supplemented with 25 mM HEPES pH 7.4, 26 mM NaHCO_3_, 2% dextrose, 15 mg/L hypoxanthine, 25 mg/L gentamycin, and 0.5% Albumax II. Incubator conditions remained at 37°C and 5% CO_2_, 5% O_2_, 90% N_2_, or 95% air. For comparison screening using serum vs Albumax II, culture media was changed from RPMI with 0.5% Albumax II to RPMI with 10% human serum and maintained for a minimum of 2 weeks prior to assay use.

Parasites grown in the Winzeler laboratory for screening and drug selections were maintained in RPMI 1640 medium supplemented with 0.25% Albumax I, 26 mM sodium bicarbonate, 0.1 mM hypoxanthine, and 50 μg/L gentamicin. Human O+ whole blood obtained from the Blood Bank of The Scripps Research Institute was used for cultivation (human subjects IRB number: 125933). Blood-stage cultures were kept at 37°C with 5% CO_2_, 3% O_2_, and 92% N_2_. Monitoring of parasitemia and parasite morphology was performed using microscopic evaluation of thin blood smears that were first fixed with methanol, and then stained with Giemsa.

HepG2 cells are grown at 37°C in DMEM with 10% FBS, 1% Antibiotic/Antimycotic, 0.3% NaHCO_3_, 5 mM HEPES pH 7.4, and 2 mM L-glutamine as needed in an atmosphere containing 5% CO_2_.

### Method details

#### Correlation matrix and principal component analysis

For the 57 peptaibols discussed, 48 different chemical identifiers were calculated and compared, including the octanol-water partition coefficient (*miLogP*), *AA-length*, *molecular weight*, *intrinsic complexity*, *complexity/atom*, the *EC*_*50*_ *values* in *HepG2* and *Dd2*, the *selectivity indexes*, the *percent hemolysis* at 25 μM, the *number of H-bond donors* and *acceptors*, the *log D*, *pKA*, *CNS-MPO* score, total polar surface areas (*TPSA*), *fsp3*, *heavy atom count*, number of rotatable bonds (*nrotb*), *percentages* of *C*, *H*, *N*, and *O*, the number of atoms (*natoms*), ion channel modulator capacity (*ICM score*), the number of ON groups (*nON*), the number of OHNH groups (*nOHNH*), the predicted *log P*, predicted *log S*, *number of Lipinski violations*, the *percentage* of *Aib*, *Gly*, *Ala*, *Gln*, *Val*, *Leu*, *Pro*, *Iva*, *Asn*, *Ser*, and *Ile* groups, the presence of a *leuol*, *phenol*, or *amide* end, *percentage* of *hydrophobic*, *polar*, or *nonpolar side chains*, and the classifications of *peptaibol* vs *lipopeptaibol*. The variables: *intrinsic complexity* and *complexity per atom* were determined using the Böttch Score calculator from forlilab.org.^58^ The *miLogP*, *nON*, *nOHNH*, *nrotb*, natoms, and ion channel modulator capacity were calculated using molinspiration.^59^ The HepG2 and Dd2 EC_50_s, selectivity indexes, and percent hemolysis at 25 μM were determined experimentally as discussed elsewhere. In the instance a compound exhibited a greater than (>) value, the maximum numerical value measured was substituted for calculation purposes (i.e., > 25 becomes 25). The percentages of C, H, N, or O, the heavy atom count, TPSA, CNS-MPO score, pKA, H-bond donors/acceptors, number of Lipinski violations, predicted log D, and predicted log S were determined using CDD Vault (Burlingame, CA., www.collaborativedrug.com). Values were then compared in a correlation matrix in GraphPad Prism version 9.1.1 using a two-tailed Pearson correlation with 95% confidence intervals.

Based on relevancy and impact on HepG2 EC_50_, Dd2 EC_50_, selectivity index, or hemolysis, as determined by the Pearson correlation (p < 0.05, Pearson r > |0.3|), 27 chemical identifiers were then modeled using a principle component analysis as seen in Figure S1A. Modeling was performed in GraphPad Prism version 9.1.1. Data were scaled to have a mean of 0 and a SD of 1, and the top 2 PCs with the greatest eigen values (PC1: 14.9, PC2: 5.47) were selected for comparison; showing a cumulative proportion of variance score of 75.46%. A simplified PCA showing just the loading scores for selectivity index, HepG2 EC_50_, hemolysis, and Dd2 EC_50_ was created for Figure 1B. A full list and correlation matrix of the final 27 chemical identifiers can be seen in Figure S1B.

#### Phenotypic screen

Inhibitor EC_50_ determination in 3D7 and Dd2 was performed as discussed in,^35^ based on established protocols.^60^ Assays utilized asynchronous culture at 1% parasitemia, 1% hematocrit. Culture was incubated with compound dilutions for 72 h followed by freezing at −80°C to promote lysis, then thawing and incubation for 45 min–1 h with 1× SYBR Green I in a lysis buffer (20 mM Tris-HCL, 0.08% saponin, 5 mM EDTA, and 0.8% Triton X-100). Fluorescence was read 485 nm excitation, 530 nm emission on a Synergy Neo2 multimode reader (BioTek Winsooki, VT) and EC_50_ curves were generated using CDD Vault (https://www.collaborativedrug.com/).

Cross resistance screening was performed with 0.3% parasitemia, 2.5% hematocrit, dispensed into 1536-well black, clear bottom plates with pre-spotted compound using a MultiFloTM Microplate dispenser (BioTek) at a volume of 8 μL/well. Plates where then incubated at 37°C for 72 h, after which 10× SYBR Green I (Invitrogen) in a lysis buffer (20 mM Tris/HCl, 5 mM EDTA, 0.16% (w/v) saponin, 1.6% (v/v) Triton X-) was then added using a MultiFloTM Microplate dispenser (BioTek) at a volume of 2 μL/well. After 24 h incubation at room temperature, viability was measured via fluorescence using the EnVision® Multilabel Reader (PerkinElmer) (485 nm excitation, 530 nm emission). IC_50_ values were determined in CDD vault (https://www.collaborativedrug.com/).

Screening of HZ NPDG-I resistant clones was performed at 1% parasitemia, 0.2% hematocrit with flow cytometry in V-shaped 96-well plates. Briefly, compounds were subjected to a 3-fold serial dilution with the highest concentration set at 4 μM or 8 μM. Eleven dilution points were included in addition to no-drug controls. Three technical replicates were set up for each compound at each dilution point. The Dd2-B2 parent line, or the clonal line 1B2, 2B3, 3A3, or 1E2 were added at 0.4% hematocrit and 1% parasitemia to the diluted compounds at a 1:1 ratio. The plate was incubated at 37°C for 72 h in a chamber filled with a hypoxic gas mixture (3% oxygen, 5% carbon dioxide, and 92% nitrogen). After incubation, a blood pellet was formed at the bottom of each well of the 96-well plate. Supernatant was removed without disturbing the pellet, and 20 μl of SYTO 61(1:10,000 dilution; Thermo Fisher Scientific, Cat No. S11343) in PBS was added to each well and incubated for 15 mins at room temperature. The cells in each well were then diluted with 180 μl of PBS and subjected to flow cytometry for parasitemia determination using the HTS (High-Throughput Sampler) of BD FACSCanto II (BD Biosciences, NJ). Flow cytometry data was analyzed using FlowJo Version 10.4.0. Dose response curves were generated and IC_50_ values obtained with GraphPad Prism Version 9.1.1.

#### Human cell culture conditions and cytotoxicity assay

The cytotoxicity assay was performed as reported previously.^35^ HepG2 cells were seeded at ∼2,250 cells per well 24 h prior to compound addition. Cells were then incubated for an additional 48 h prior to the addition of MTS and absorbance reading at 490 nm on Synergy Neo2 multimode reader (BioTek, Winsooki, VT). Cytotoxicity EC_50_ values were determined using CDD Vault (https://www.collaborativedrug.com/).

#### *P. berghei* liver stage assay

The liver stage was performed as discussed in.^61^ Liver-stage inhibitory activity was evaluated in HepG2-A16-CD81 cells and using *P. berghei* sporozoites (*P. berghei* ANKA GFP-Luc-SMcon) freshly obtained by dissecting salivary glands of infected *A. stephensi* mosquitoes. Cells were first seeded in 1536-well plates (Greiner Bio) containing 50 nL of test and control compounds dissolved in DMSO and incubated for 24 h. Sporozoites were then added to each well at a density of 1 × 10^3^ cells per well and kept at 37°C for 48 h. Afterwards, luciferin reagent (Promega BrightGlo) was added to each well, and luciferase activity was detected using a Perkin Elmer Envision plate. As a counter screen, HepG2-A16-CD81 cells were seeded as above and exposed to drug in the absence of sporozoites. Viability was evaluated by adding Promega CellTiterGlo® followed by luminescence measurement using a Perkin Elmer Envision plate reader. IC_50_ values were determined using CDD vault (https://www.collaborativedrug.com/).

#### High content imaging *P. berghei* assay

HepG2 cells infected with luciferase-expressing *P. berghei* sporozoites were seeded onto compound-loaded 384-well plates 2 hours after sporozoite addition. A live luciferase readout at 48 HPI^62^ quantifies compound activity against parasite biomass, while HCI of MSP1 and AMA1 expression at 72 HPI quantifies effects on liver stage maturation and hepatic merozoite formation. Compound effects on hepatocytes are assessed based on HepG2 area occupied in the 72 HPI HCI assay. All data are normalized to in-plate DMSO controls.

#### Percent hemolysis determination

Hemolysis assay performed as outlined in Duvall et al. with some modifications.^63^ Whole blood was first washed and resuspended in PBS to isolate RBCs. Compound was then added in serial dilutions to PBS with 1% hematocrit, with vehicle or 0.01% saponin used as controls. Plates were incubated for 1 h at 37°C then centrifuged for 10 min at 900 x g. Supernatant was then transferred to a clear plate and absorbance was read at 550 nm. Extended incubation with compound (up to 3 days) was performed during assay optimization, with no changes noted in percent hemolysis.

#### Synchronous stage specificity assay

SSA was performed in triplicate as previously described.^64^ In summary, Dd2 culture was synchronized by combination of MACS column^65^ and sorbitol treatment,^66^ and cultured at 1% parasitemia, 2% hematocrit, for 54 h. Compound was added at approximately 6, 18, 30, or 42 HPI, and samples were taken every 12 h after treatment times for Giemsa staining and flow cytometric analysis. HZ NPDG-H and HZ NPDG-I were tested at 5 x EC_50_, TC HA-II tested at 3 x EC_50_ due to its higher hemolytic potential. All test values above EC_90_. Flow samples were fixed in 4% paraformaldehyde, incubated for 30 min at 37°C, then washed 3 times in PBS. Fixed samples were stored at 4°C prior to staining, then resuspended in 0.25% Trition X-100 and incubated for 10 min with shaking. Permeabilized samples were centrifuged and resuspended in a 50 μg/mL RNase solution, and incubated for 3 h at 37°C. After centrifugation, samples were resuspended in a YOYO-1 solution (500 nM final concentration) and read on a CytoFLEX S (Beckman Coulter, Brea, CA) at 488 nm excitation, with 100,000–500,000 events recorded per sample. Infected RBCs were gated using an uninfected RBC control in FlowJo version 10. After gating populations, FITC channel values were extracted from FlowJo and the sum total fluorescence of the iRBCs and the median fluorescence for each timepoint were graphed in Graph Pad Prism Version 9. Assay workflow outline was created using BioRender.

#### Killing profile

Killing profile analyses were performed as previously described,^64^ in asynchronous Dd2 culture, starting at approximately 1% parasitemia, 4% hematocrit. Compound was added to culture in majority ring stage and incubated for 12, 24, or 48 h prior to washing and sample collection. Media were then changed normally every 24 h for 6 days. Samples taken each day were used for Giemsa thin smears, and flow cytometric analysis to monitor parasitemia and mitotracker retention. For staining, samples were incubated in a solution of 1:1000 SYBR Green I and 0.6 μM Mitotracker Deep red FM for 30 min then read on a CytoFLEX S (Beckman Coulter, Brea, CA), with 100,000 events recorded per sample. Flow cytometry was gated in FlowJo Version 10 using DHA, vehicle, no Mitotracker, and uninfected RBC controls. Assay workflow outline was created using BioRender.

#### Transmission electron microscopy

For TEM imaging, samples were prepared as previously described.^67^ In summary, Dd2 strain parasites were exposed to a 5 x EC_50_ concentration of selected compounds, or DMSO control, for either 1.5, 3, 6, 12, or 24 h. Culture was collected by centrifugation and washed with PBS, pH 7.4. Samples were then fixed in 2% paraformaldehyde, 2.5% glutaraldehyde, in a 0.1 M sodium cacodylate buffer, pH of 7.4, (Electron Microscopy Sciences, Hatfield, PA) and incubated for 2 h at room temp with mild shaking. After fixation, samples were pelleted and stored cold but not frozen in 0.2 M sodium cacodylate buffer prior to staining and embedment in Eponate 12 resin (Ted Pella Inc.). Images were taken using a JEOL 1200 EX transmission electron microscope (JEOL USA Inc., Peabody MA) and processed in AMT Image Capture Engine V602 software (Advanced Microscopy Techniques, Woburn, MA.).

#### FITC-dextran measurements

Preloading of 3D7 parasites with FITC-dextran was done as outlined in Saliba et al.^68^ In summary, 2.25 mL of pre-warmed lysis buffer (5 mM HEPES, 11 mM glucose, 2 mM MgCl_2_, 2 mM ATP, and 110 μM FITC-dextran, pH 7.4) was added to 1 mL of packed RBCs and incubated at 30°C for 10 min. After, 2.25 mL of resealing buffer (280 mM NaCl, 40 mM KCl, 11 mM glucose, and 1 mM HEPES, pH 7.4) was added and cells were pelleted at 1,200 x g for 5 min then washed 3 x in RPMI. FITC-dextran loaded RBCs were then resuspended with 1 mL of synchronous trophozoite stage parasites (10–20%) parasitemia, at 4% hematocrit. Cultures were maintained in 50 mL for a minimum of 2 life cycles, until parasitemia reached >15%.

For assay use, trophozoite stage parasites were functionally isolated as described by van Schalkwyk et al. and Kirk et al. with some modifications.^69^^,^^70^ Parasites were resuspended in warm media with 0.05% saponin then centrifuged immediately at 2,060 x g for 10 min. Cells were washed in RPMI media 3 x then resuspended in HEPES buffered saline (100 mM KCl, 30 mM NaCl, 2 mM MgCl_2_, and 5 mM HEPES, pH 7.3) at 5 x 10^6^ cells/mL. Cells were incubated with either a 5 x EC_50_ concentration of peptaibols, 100 nM of concanamycin A, or a DMSO control. Samples were collected at 0.5, 1, and 2 h for flow cytometry using a CytoFLEX S (Beckman Coulter, Brea, CA), excitation 488 nm, emissions 530 and 585 nm. Gating was performed in FlowJo Version 10 using FITC-dextran loaded and unloaded controls. The ratio (R_gy_) of the fluorescent intensity values was then graphed using Graph Pad Prism Version 9. For microscopic analysis, samples were collected after 2 h of treatment and imaged on a Leica TCS SP5 II confocal microscope (Leica Microsystems, Wetzlar, DE.) at excitation 488, emission 520 nm with a 30 nm bandpass filter, then processed with LAS-X software. Significance determined using a one-way ANOVA multiple comparisons test in GraphPad Prism Version 9.0.

#### Electrophysiology

Ion channel formation by peptaibols was evaluated with reconstitution into planar lipid bilayers using the Orbit Mini workstation (Nanion). Bilayers were formed from a 5 mg/ml decane solution of 1,2-diphytanoyl-sn-glycero-3-phosphocholine (DPhPc, Avanti Polar Lipids) across 100 μm diameter orifices in the MECA 4 recording chip with 4 microcavities. The bilayers were bathed with saline (in mM: 70 NaCl, 70 KCl, 2.5 MgCl2, 2 CaCl2, 10 HEPES, 10 MES, 10 glucose, pH 7.0) at both faces, with resistances of up to 500 GΩ before channel incorporation. Peptaibols were added from a 10 mM DMSO stock to the cis chamber at a final 1 μM concentration; spontaneous incorporation into the bilayer typically occurred within 10–15 min. Recordings were acquired at 20 kHz and filtered at 1.25 kHz.

#### RNASeq analysis

*P. falciparum* 3D7 culture was synchronized as previously described^64^ by combination of MACS column (Mitenyi Biotic, Auburn, CA)^65^ and 5% sorbitol.^66^ In brief, asynchronous culture with a majority ring stage population was centrifuged, pelleted, and resuspended in 5% sorbitol and incubated at room temp for 10 min. Parasites were then washed with culture media and returned to the culture incubator. For MACS column, columns were rinsed once with RPMI prior to parasite addition. After flow-through was complete, parasites on the column were eluted onto culture plates and returned to the culture incubator. Synchronous cultures were then treated in late trophozoite (24–28 HPI) for 1 h with an EC_50_ concentration of harzianin NPDG I. This process was repeated for 3 independent biological replicates. RNA was then extracted using Direct-zol™ RNA Miniprep Plus (Zymo Research Corporation, Irvine, CA.) following manufacturers protocols. In brief, culture was lysed with 0.1% saponin and incubated for 5 min prior to centrifuging at 900 x g for 6 min at 4°C. Cells were washed once with RPMI then once with PBS before resuspension in triazole reagent. Samples were mixed vigorously for 5 min, centrifuged at 16,000 x g for 1 min, then transferred to a RNase free tube, and an equal volume of EtOH was added. After mixing, sample was loaded onto column and centrifuged at 16,000 x g for 30 sec then washed with pre-wash buffer. Columns were incubated with DNase buffer for 15 min then washed with pre-wash buffer. Two more wash steps were performed before allowing any excess EtOH to dry for 2 min. Finally, columns were transferred to a new RNase free tube in which RNA was eluted with RNase free water.

Preliminary integrity and concentration determination was performed via nanodrop and RNA gel electrophoresis, prior to a full integrity check via Agilent Technologies 2100 Bioanalyzer. After rRNA depletion, RNA fragmentation was performed using elevated temperatures and divalent cation buffers. Library preparation was done using the Illumina TruSeq-stranded-total-RNA-sample preparation protocol, with Agilent Technologies 2100 Bioanalyzer High Sensitivity DNA Chip for quality control, and Illumina’s NovaSeq 6000 sequencing system for paired-ended sequencing. For transcript assembly, Cutadapt^71^ and LC Sciences in house perl scripts were used to remove low quality or undetermined bases and reads, along with adaptor contamination. Sequence quality was then confirmed using FastQC.^72^ Reads were mapped to the *P. falciparum* genome using Bowtie2^73^ and HISAT2,^74^ then assembled using StringTie.^75^ To assist in the filtering process, the transcriptomes from HZ NPDG-I and the vehicle control were assembled simultaneously to construct a merged transcriptome using perl scripts and gffcompare.^76^ Finally, StringTie^75^ and edgeR^77^ were used to estimate transcript expression levels by calculating FPKM using the following equation:

$$FPKM=\frac{total\;exon\;fragments}{mapped\;reads\left(millions\right)\;x\;exon\;length\left(kB\right)}$$

To calculate p value, a parametric F-test comparing nested linear models was performed using edgeR.^77^ The q-values were also calculated using a Benjamini-Hochberg procedure. Differential expression for mRNA was calculated at the gene level (all transcript isoforms grouped as 1 gene) and the transcript level (all transcript isoforms ranked individually). To identify novel lncRNA, all transcripts shorter than 200 bp or matching known mRNAs or lncRNAs were first removed. The coding potential of the remaining transcripts was then determined using CPC^78^ and CNCI,^79^ and transcripts with a CPC score <0.5 and/or CNCI score <0 were removed. The remaining transcripts were then categorized into groups *i* (intronic transfrag), *j* (potentially novel fragment/isoform with one or more splice junction shared with the reference transcript), *o* (generic exonic overlap with reference), *u* (unknown intergenic), or *x* (antisense exonic overlap). The predicted *cis-*targets of the novel lncRNAs were then determined for the coding genes 100,000 bp upstream and downstream with perl script.

Hierarchical clustering and *Z* score transformation for heatmaps was performed using Instant Clue^80^ of significant (p < 0.05) differentially expressed transcripts. Results were imported into Graph Pad Prism Version 9 for graphing and outlier removal. GO-analysis of significant (p < 0.05) transcripts identified with gene-level sorting was performed using Revigo.^81^ Node color corresponds to log10(p value) of GO-term, node size corresponds to the log10(number of annotations of GO-term in *P. falciparum* in the EBI GO database). Nodes are grouped based on semantic similarity.

#### Real-time quantitative PCR

For RT-qPCR validation, total RNA was extracted as previously described from vehicle and HZ NPDG-I treated samples collected for the RNASeq analysis. A total of 500 ng of RNA from each sample was reverse transcribed into cDNA using the SuperScript First-Strand Synthesis System for RT-PCR (Invitrogen, Waltham, MA) with 100 ng of random hexamers. Synthesized cDNA from each of the 3 biological replicates was tested using Select Master Mix (Life Technologies, Carlsbad, CA) with 400 nM of forward and reverse primers using a QuantStudio 7 Flex qPCR machine (Thermo Fisher, Waltham, MA). SYBR fluorescence was normalized to passive reference dye ROX. C_t_ values were normalized using the 2^ΔΔCt^ method by normalizing to housekeeping gene serine-tRNA ligase (PF3D7_0717700) and then normalizing between the treated and untreated controls. PCR parameters included 50°C for 2 min, 95°C for 10 min, followed by 40 cycles of 95°C for 15 s, 55°C for 30 seconds, and 60°C for 30 seconds. Primer pair sequences are listed in full in Table S5.

#### β-hematin inhibition assay

Inhibition of β-hematin was determined as previously described with some modifications.^82^ Serial dilutions of peptaibols, CQ, or DMSO vehicle were added to microtiter plates with 1 M propionate buffer (pH 5.2), 0.5 mg phosphatidylcholine (Sigma-Aldrich, St. Louis, MO), and 2 mM hemin in 0.1 M NaOH. Assay plates were incubated at 37°C with gentle shaking for 16 h. A combination of 7.5% SDS in a 0.1 M bicarbonate buffer (pH 9.1) was then added to microtiter wells at a 1:3 ratio with gentle mixing. Plates were incubated for 10 min to allow crystals to settle before taking 50 μL of supernatant and adding it to 200 μL of 2.5% SDS in a 0.1 M bicarbonate buffer, pH 9.1. After mixing, absorbances were read at 405 nm on a Synergy Neo2 multimode reader (BioTek, Winsooki, VT). Results were normalized using 200 μM chloroquine and vehicle controls, and graphed in Graph Pad Prism Version 9. Images of the 200 μM treatment or control prior to SDS quenching were taken using a Nikon Eclipse TE200 inverted microscope.

#### The *in vitro* evolution of drug-resistance against harzianin NPDG I

Continuous cultivation of asexual blood-stage parasites was performed under standard conditions as previously described.^57^ Parasites were grown in human O+ whole blood obtained from the Blood Bank of The Scripps Research Institute (TSRI) (La Jolla, CA). Parasitemia and parasite morphology were assessed using a microscopic evaluation of thin blood smears that were first fixed with methanol (Merck) and then stained with Giemsa (Sigma).

A clone of the Dd2 strain (Dd2-B2) of *P. falciparum* was used for *in vitro* evolution of resistance against harzianin NPDG I. Three flasks of 1.2 x 10^8^ parasites were continuously exposed to a stepwise-increasing concentration of the compound over ∼3 months starting from 20 nM up to a final concentration of 100 nM. Once a stable rightward shift in 72 h IC_50_ was observed, cultures were subjected to limiting dilution to obtained harzianin-resistant clones. Each clone was expanded, phenotyped to confirm resistance, and submitted for whole genome sequencing. An untreated flask of Dd2-B2 parasites was maintained in parallel throughout the course of *in vitro* selection as a control.

#### Whole genome analysis

RBC-free parasites were first obtained by lysing infected cells with 0.05% saponin in 1 x PBS. Genomic DNA was then extracted using a DNeasy Blood and Tissue Kit (Qiagen) according to manufacturer’s instructions. Sequencing libraries were prepared with the Nextera XT kit (Cat. No FC-131-1024, Illumina) via the standard dual index protocol and sequenced on the Illumina NovaSeq 6000 S4 flow cell to generate paired-end reads 100bp in length. Reads were aligned to the *P. falciparum* 3D7 reference genome (PlasmoDB v13.0) using the previously described pipeline.^41^ A total of 9 clones were sequenced to an average coverage of 244x, with an average of 85% of reads mapping to the reference genome. Following alignment, SNVs and INDELs were called using GATK HaplotypeCaller and filtered according to GATK’s best practice recommendations.^83^ Variants were annotated using a custom SnpEff database and only calls present in the resistant clone but not the sensitive parent clone were considered. CNVs were identified by differential log2 copy ratio as described in the GATK 4 workflow. Briefly, read counts were collected across genic intervals for each sample. Copy ratios were calculated after denoising read counts against a strain-matched panel of normals composed of non-drug-selected Dd2 parasite samples.

#### Plasmid construction and parasite transfection

A conditional knockdown parasite line was generated for the *P. falciparum* multi-drug resistance 1 (MDR1; PF3D7_0523000) following previously described procedures.^84^^,^^85^ The native PfMDR1 locus was engineered using CRISPR/*Sp*Cas9 to afford transcriptional fusion of 10 tandem TetR-DOZI binding aptamers in the 3′UTR for anhydrotetracycline (aTc)-dependent regulation of *Pf*MDR1 expression.

To construct the donor vector, the following were prepared: a) PCR-amplified right homology region (RHR); b) synthetic fragment comprised of a left homology region (LHR) fused to a recodonized region at the 3′-end of the gene (stop codon excluded) to eliminate the *Sp*Cas9 target site in the edited locus; and c) synthetic fragment encoding the single guide RNA (sgRNA) for *Sp*Cas9-mediated editing. Synthetic fragments were prepared on the BioXP™ 3200 (Codex). All primer and synthetic fragment sequences are included in the key resources table. These fragments were combined by Gibson assembly into the pSN054 base vector, which includes preinstalled V5-2xHA epitope tags, a 10x tandem array of TetR aptamers upstream of an *Hsp86* 3′UTR, and a multicistronic cassette for expression of TetR-DOZI (regulation), *blasticidin S-deaminase* (selection marker) and a *Renilla luciferase* (*RLuc*) reporter. The LHR and recoded region was installed in-frame with tandem V5-2x-hemagglutinin (HA) tags to afford *C*-terminal epitope-tagged *Pf*MDR1 post-editing, and upstream of the regulatory aptamer array. The Sanger sequence verified and restriction mapped final donor vector was maxi-prepped, transfected into an NF54::pCRISPR line expressing *Sp*Cas9 and T7 RNA polymerase using the red blood cell pre-loading method.^86^ Parasite cultures were maintained continuously in 500 nM anhydrotetracycline (aTc, Sigma-Aldrich 37919) and drug selection with 2.5 *μ*g/mL of Blasticidin S (RPI Corp B12150-0.1) was initiated 4 days after transfection. Cultures were monitored by Giemsa smears and RLuc measurements.

#### Transfectant line growth assay

Assessment of parasite proliferation rate in the presence and absence of aTc was carried out using luminescence as a readout of growth. In 96-well U-bottom BD Falcon™ plates, synchronous ring-stage parasites were set up in triplicate and cultured in the presence (0.5 μM) and absence of aTc and luminescence measured at 0 and 72 h by quantitating luminescence using the Renilla-Glo(R) Luciferase Assay System (Promega E2750) and the GloMax® Discover Multimode Microplate Reader (Promega). Parasite growth was determined from normalized RLuc values, with dihydroartemisinin- (DHA) treated (0.5 *μ*M) samples as a no growth control. Data were analyzed using GraphPad Prism Version 8.

#### MDR1 compound susceptibility assays

Stock solutions of compounds were serially diluted to yield final in-assay concentrations ranging from 0.005-2.8 *μ*M for harzianin NPDG H and alamethicin, 0.0006-0.312 *μ*M for harzianin NPDG I, 0.008-4 *μ*M for trichorzin HA II, and 0.05-0.00009 *μ*M for mefloquine (positive control). As a control cell line, a parasite line expressing an aptamer-regulatable fluorescent protein integrated in the *cg6* locus^84^ was assayed in parallel. Synchronous ring-stage parasites in the presence (0.5 *μ*M) and absence of aTc were dispensed into 384-well polystyrene microplates (Corning®), and compounds added using the Janus® platform (PerkinElmer). DMSO- and DHA (0.5 *μ*M) served as normalization controls for maximal and minimum signal. Luminescence was measured after 72 h and IC_50_ values were obtained from normalized dose-response curves using GraphPad Prism Version 8.

### Quantification and statistical analysis

For the principal component analysis, variables were compared using a correlation matrix in GraphPad Prism version 9.1.1 using a two-tailed Pearson correlation with 95% confidence intervals. Non redundant variables related to HepG2 EC_50_, Dd2 EC_50_, or hemolysis with a Pearson r > |0.1|, p < 0.05 were used to generate the final principal component analysis. The top principal components with the grated eigen values were chosen for modeling. The percent hemolysis, HepG2 EC_50_, Dd2 EC_50_, 3D7 EC_50_, β-hematin inhibition, RT-qPCR and FITC-dextran measurements, were all performed in biological triplicates. Significance for the FITX-dextran measurements was determined using a one-way ANOVA multiple comparisons test in GraphPad Prism Version 9.0. Each biological replicate contained 3 technical replicates for each concentration data point, including those for 3 0% controls and 3 100% controls. The Z′-factor was determined for each of these assays using the mean and standard deviations of the controls. Only assays with a Z-factor greater than 0.7 were used. The killing profile, luciferase one liver stage assays, SSA, RNASeq assays were also performed with three biological replicates. The p value for the RNASeq was calculated using a parametric F-test comparing nested linear models with edge R. The q-values were then calculated using a Benjamini-Hochberg procedure. Gene ontology p values were calculated in PlasmoDB using a Fishers exact test. High content imaging liver stage assays were performed in biological duplicate or triplicate, as indicated.

## Supplemental information

Document S1. Figures S1–S5 and Tables S1 and S2

Table S3. RNA sequencing results for HZ NDPG-I treated culture

Table S4. HZ NPDG-I resistance line sequencing

Table S5. List of primers used for RT-qPCR

Data S1. Spectra and purify information for new and primary peptaibols

Document S2. Article plus supplemental information
