# Supplementary material for: Antiplasmodial peptaibols act through membrane directed mechanisms
Source: Cell Chem Biol. 2024 Feb 15;31(2):312–325.e9. doi: 10.1016/j.chembiol.2023.10.025 (PMC10923054; doi:10.1016/j.chembiol.2023.10.025)
Supplement: Document S1. Figures S1–S5 and Tables S1 and S2 [file mmc1.pdf]

Cell Chemical Biology, Volume 31

Supplemental information

Antiplasmodial peptaibols act through membrane directed mechanisms

Jennifer E. Collins, Jin Woo Lee, Frances Rocamora, Gagandeep S. Saggu, Karen L. Wendt, Charisse Flerida A. Pasaje, Sebastian Smick, Natalia Mojica Santos, Raphaella Paes, Tiantian Jiang, Nimisha Mittal, Madeline R. Luth, Taylor Chin, Howard Chang, James L. McLellan, Beatriz Morales-Hernandez, Kirsten K. Hanson, Jacquin C. Niles, Sanjay A. Desai, Elizabeth A. Winzeler, Robert H. Cichewicz, and Debopam Chakrabarti

Supplemental Information

Supplemental Figures S1-S5

Supplemental Tables S1-S2

Supplementary Figures

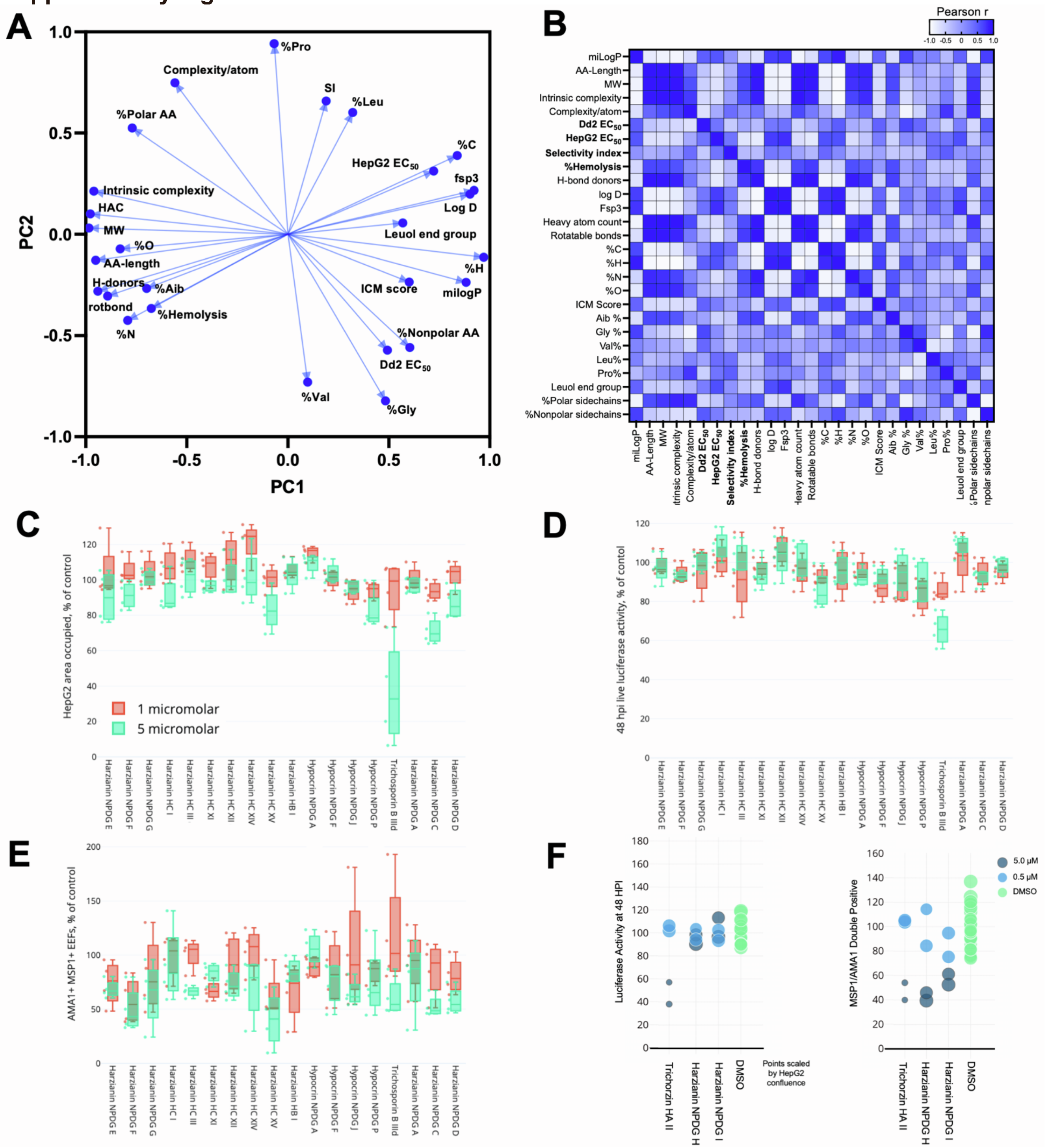

Figure S1. **Expanded PCA and liver stage profiling. Related to Figure 1.**

(A) Full PCA of final 27 loadings used.

(B) Correlation matrix of final 27 identifiers and corresponding Pearson r scores.

(C) Liver stage HCI assay readout for HepG2 area occupied. Compounds tested at 1 and 5 µ; n = 4 experiments, with each point representing the mean of 2 technical replicates.

(D) Liver stage HCI assay readout for luciferase activity at 48 HPI.

(E) Liver stage HCI assay readout for number of AMA1+ MSP1+ extra erythrocytic form (EEF) parasites.

(F) Liver stage HCI assay readout for select *T. harzianum* peptaibols tested at 5 and 0.5 μM.

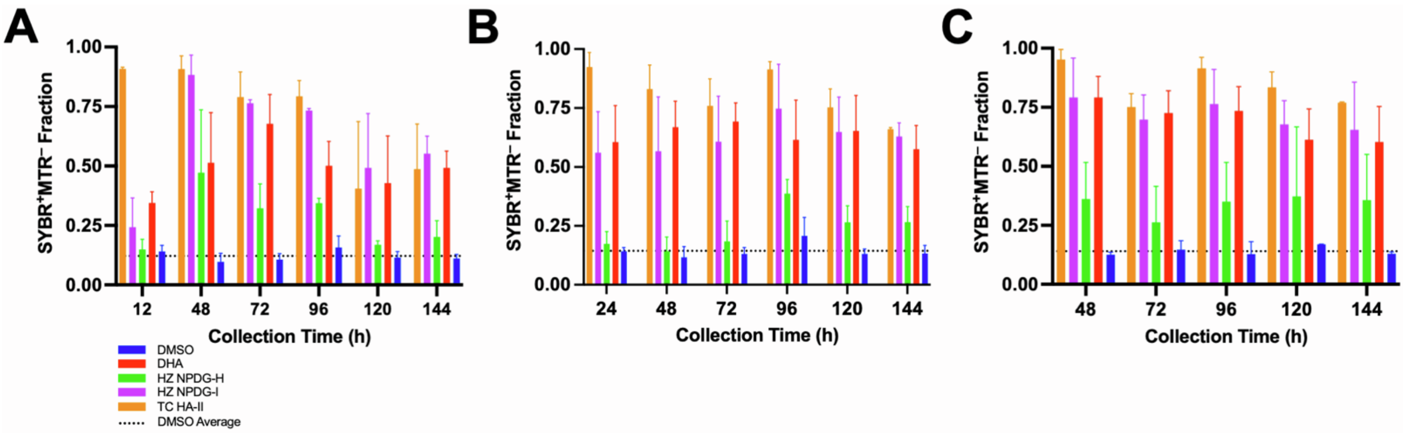

Figure S2. Additional killing profile staining of *T. harzianum* peptaibols. Related to Figure 2.

(A) Mitotracker negative parasite fraction after 12 h of compound exposure. Peptaibols added at 10 x EC_{50}. Dotted line represents DMSO average across all collection times. DHA used as a fast-acting control. Data represent mean ± SEM of 3 biological replicates.

(B) Mitotracker negative parasite fraction after 24 h of compound exposure.

(C) Mitotracker negative parasite fraction after 48 h of compound exposure.

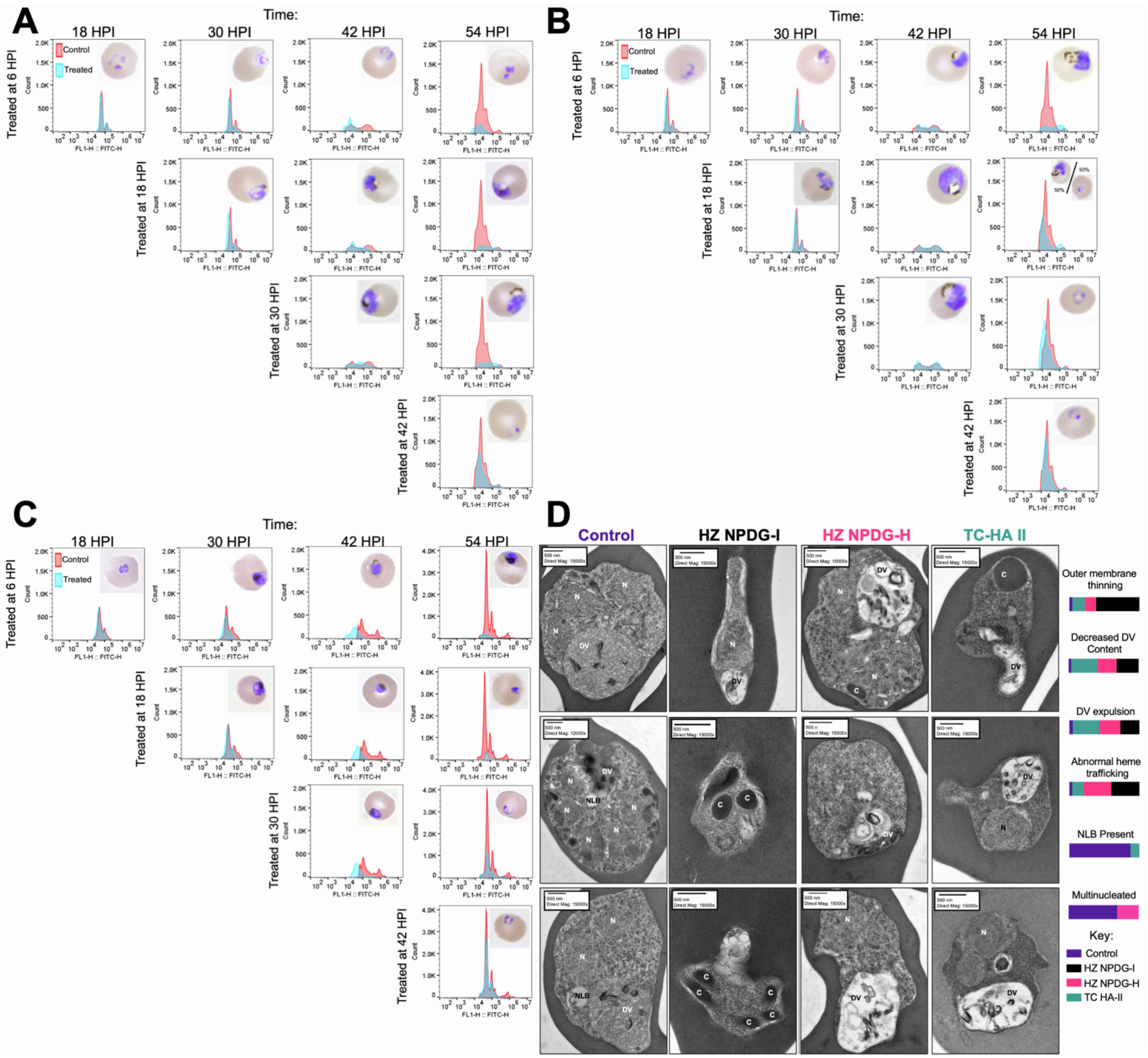

Figure S3. Addition SSA and TEM images of select *T. harzianum* peptaibols. Related to Figure 3.

(A) Flow cytometric analysis and Geimsa staining of treated culture following addition at approximately 6, 18, 30, or 42 HPI with HZ NPDG-I at 5 x EC_{50}. 500,000 flow events recorded per timepoint.

(B) SSA with HZ NPDG-H at 5 x EC_{50}.

(D) SSA with TC HA-II 3 x EC_{50} due to hemolytic potential.

(E) Additional TEM images for TC HA-II, HZ NPDG-I, and HZ NPDG-H. N = nucleus, DV = digestive vacuole, C = cytostome, NLP = neutral lipid body. Includes color coded breakdown of peptaibol treatments. Full length of bar represents the total fraction of parasites imaged. Color represents condition type with the colored portion per bar representing the fraction of images in that condition displaying the listed parasite morphology.

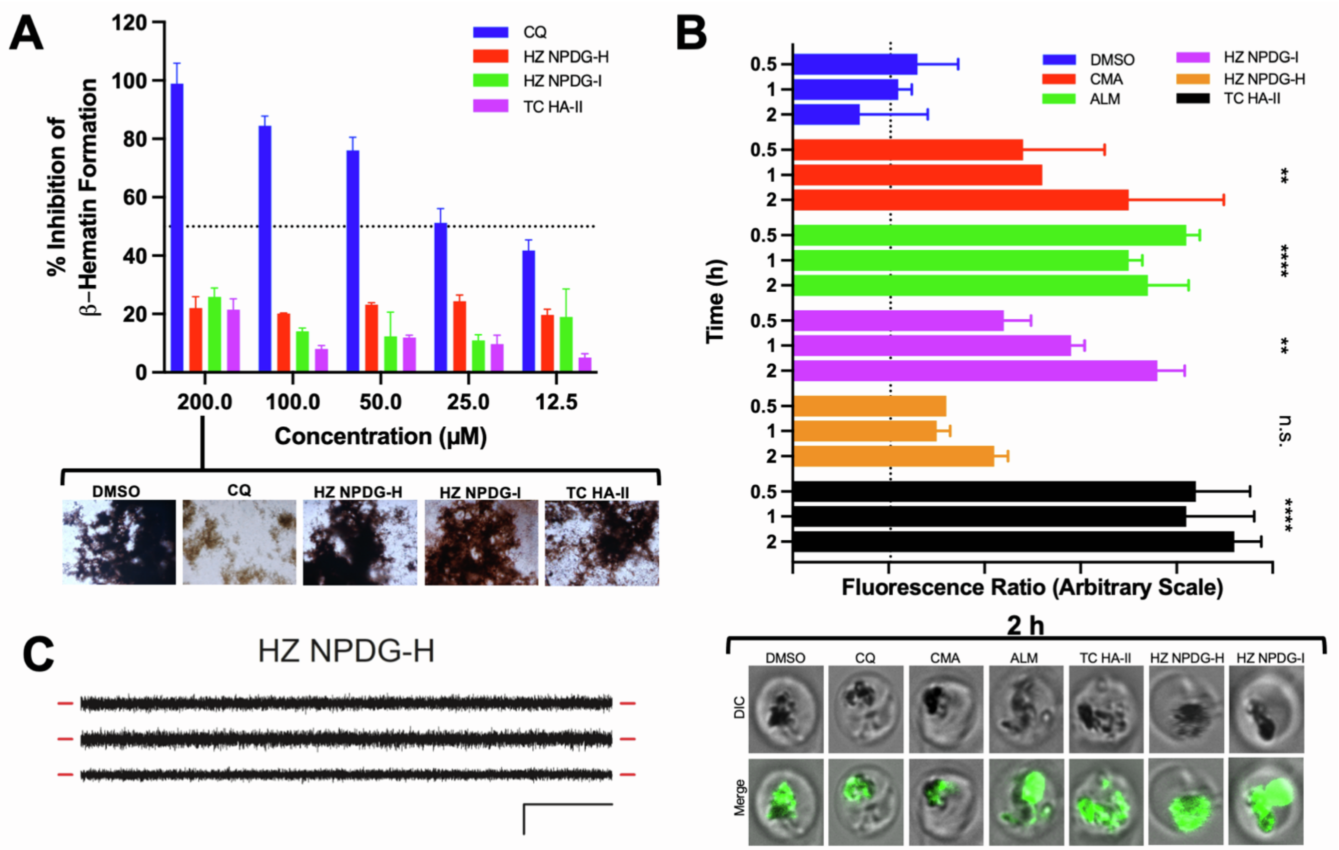

Figure S4. Additional impact of *T. harzianum* peptaibols on DV functions. Related to Figure 4.

(A) Inhibition of β-hematin crystal formation. Dotted line represents 50% inhibition of β-hematin. Images taken following treatment with 200 µM of the respective compounds or vehicle. Values represent mean ± SEM of 3 biological replicates.

(**B**) Fluorescence ratio of isolated parasites treated for 0.5, 1, or 2 h with 5 x EC_{50} of peptaibols, 100 nM concanamycin A (CMA), or DMSO vehicle control (ALM: alamethicin). Dotted line represents DMSO average across all times. Images represent FITC-dextran localization at final time of 2 h. Values represent mean ± SEM of 3 biological replicates.

(C) Current recordings from 3 separate bilayers after addition of 1 µM HZ NPDG-H. Red dashes represent baseline currents. The absence of deviations from this baseline suggests that this peptaibol does not produce detectable channel activity under these recording conditions (n = 8 bilayers). Scale bar, 500 ms (horizontal)/5 pA (vertical). Imposed bilayer potentials (V_b), -60 mV for all traces.

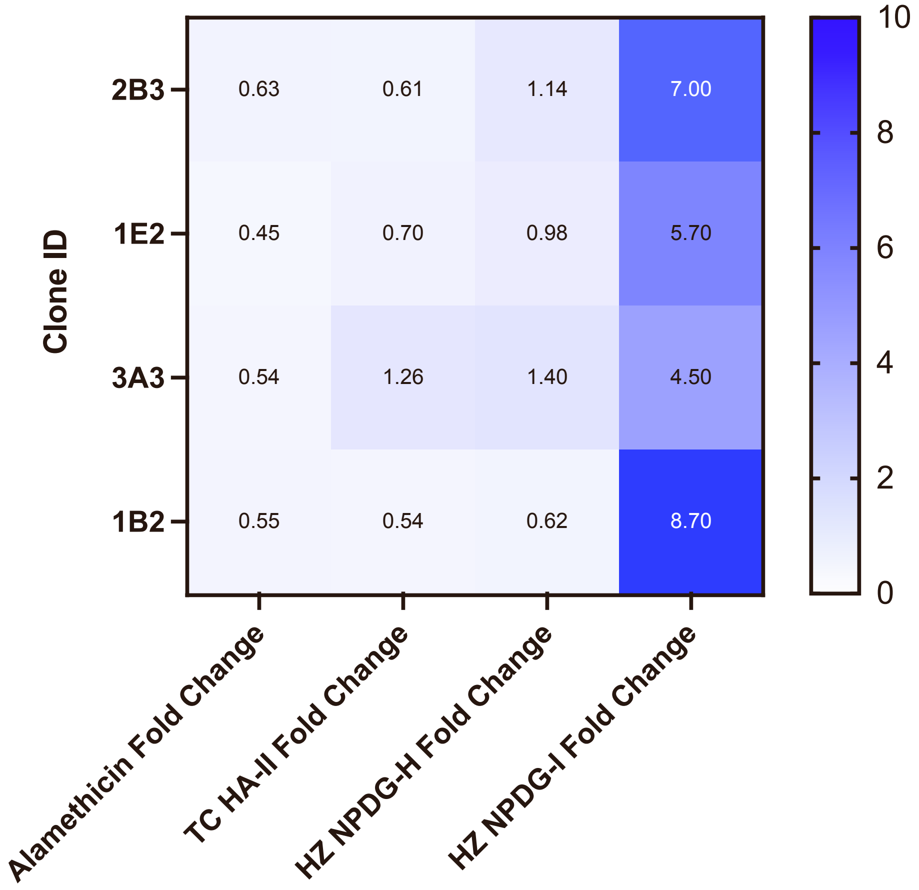

Figure S5. Activity analysis of peptaibols in HZ NPDG-I resistant clones. Related to Figure 6. Four of the clones generated through the *in vitro* evolution of resistance to HZ NPDG-I were screened against the parental line. The fold change in IC_{50} was then compared to the shift seen in HZ NPDG-I.

Supplementary Tables S1-S2

Table S1. Harzianin NPDG I cross resistance screening. Related to Figure 1.

**Asexual Blood Stage**				
**Dd2 EC$_{50}$ (μM)**	**PfACS_A597 EC$_{50}$ (μM)**	**PfCARL_I1139K EC$_{50}$ (μM)**	**PfPI4K_S1320L EC$_{50}$ (μM)**	**RIs**
0.013 ± 0.006	0.023 ± 0.005	0.008 ± 0.001	0.013 ± 0.003	0.62-1.8
**Asexual Blood Stage DHODH**		**Liver Stage**		
**Attb EC$_{50}$ (nM)**	**attb ScDHODH EC$_{50}$ (nM)**	**PbLuc EC$_{50}$ (μM)**	**HepG2 EC$_{50}$ (μM)**	
5.7 ± 2.3	9.1 ± 1.5	2.7 ± 0.6	> 50	

Values represent mean ± SEM of 3 biological replicates.

Table S2. Antiplasmodial activity with Albumax II or serum media. Related to Figures 3 and 4.

ID	Albumax II EC_{50} (μM)	Serum EC_{50} (μM)	Fold Change[a]
CQ	0.16 ± 0.01	0.10 ± 0.02	1.6
HZ NPDG-H	0.33 ± 0.09	0.69 ± 0.09	0.48
HZ NPDG-I	0.10 ± 0.01	0.26 ± 0.03	0.38
TC HA-II	0.45 ± 0.06	2.2 ± 0.27	0.20

Notes: Results expressed as mean ± SEM from 3 biological replicates.
[a] Fold Change = Albumax II EC_{50} / Serum EC_{50}
