# Supplementary material for: Antiplasmodial peptaibols act through membrane directed mechanisms
Source: Cell Chem Biol. 2024 Feb 15;31(2):312–325.e9. doi: 10.1016/j.chembiol.2023.10.025 (PMC10923054; doi:10.1016/j.chembiol.2023.10.025)
Supplement: Data S1. Spectra and purify information for new and primary peptaibols [file mmc5.pdf]

Data S1. UPLC-MS spectra and ^{1}H and ^{13}C NMR data for peptaibols and purity information for alamethicin. Related to the STAR Methods.

Ac-D-Iva-L-Gln-L-Leu-L-Ile-Aib-L-Pro-D-Iva-L-Leu-Aib-L-Pro-L-Leuol

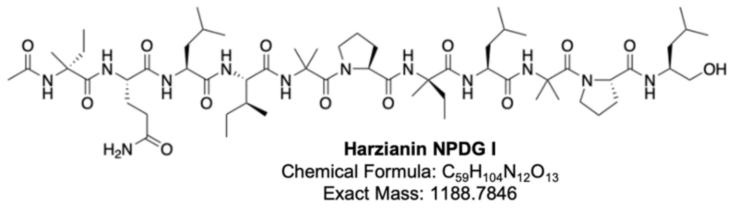

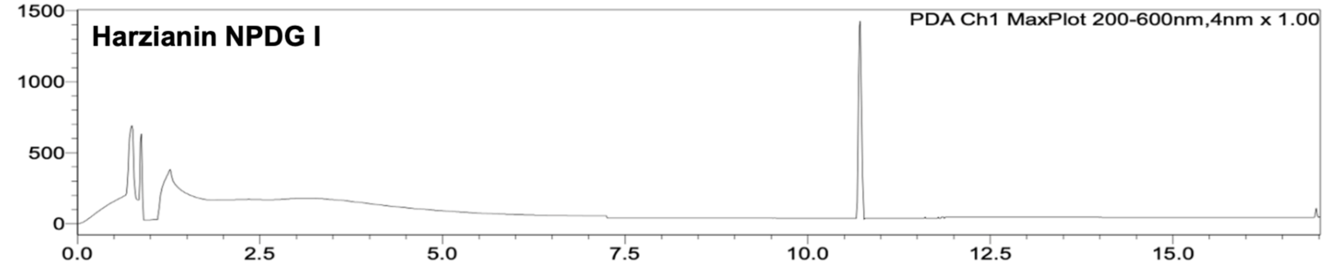

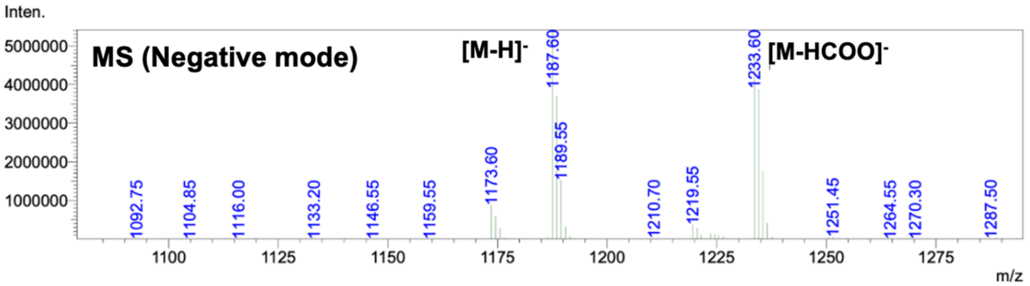

HPLC condition
C_{18} ODS column
10% ~100% ACN in 13 min
100% ACN 13 ~ 15 min

UPLC-MS spectra of harzianin NPDG I

Ac-D-Iva-L-Gln-L-Leu-Aib-L-Pro-L-Ala-L-Ile-Aib-L-Pro-D-Iva-L-Leu-Aib-L-Pro-L-Leuol

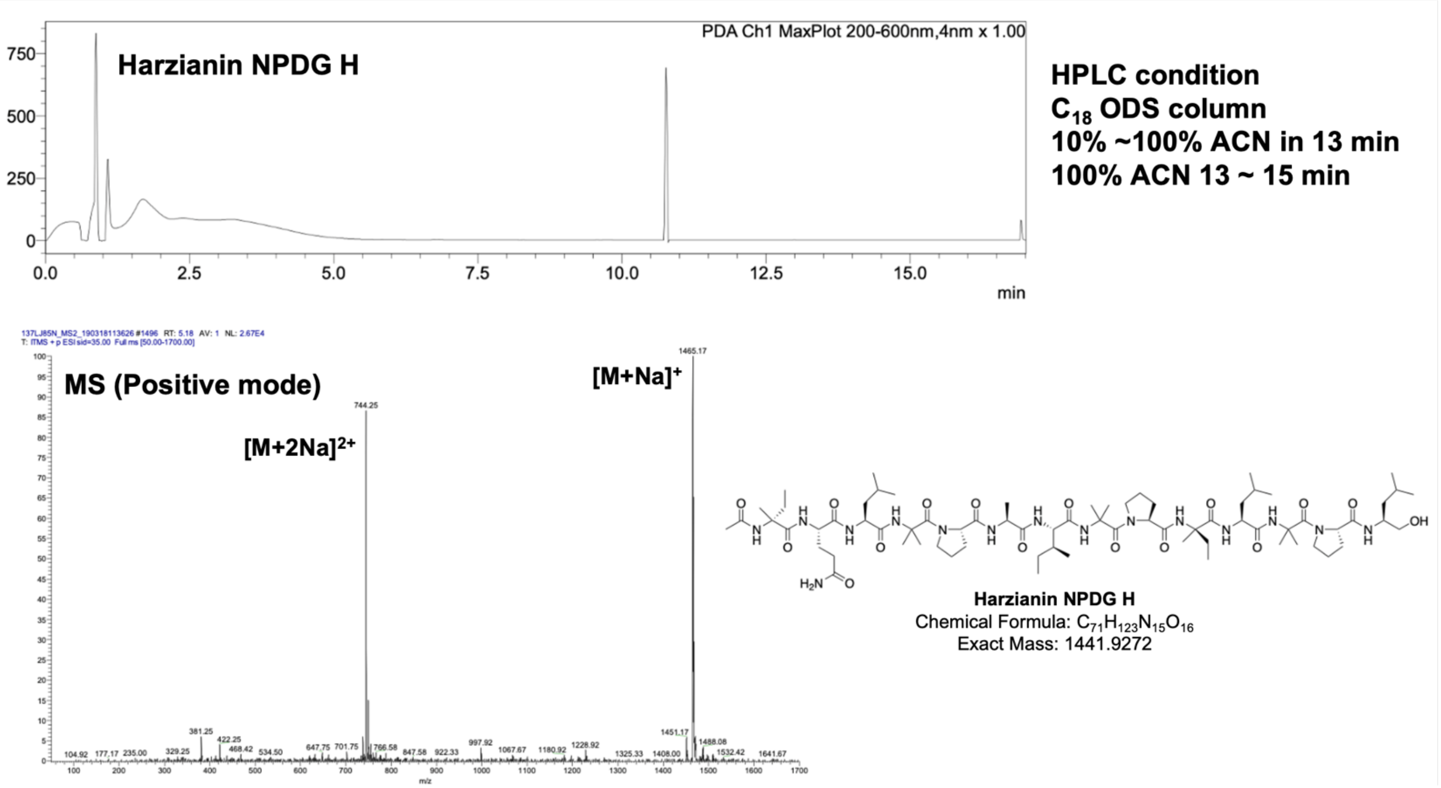

UPLC-MS spectra of harzianin NPDG H

Ac-Aib-Gly-L-Ala-Aib-Aib-L-Gln-Aib-L-Val-Aib-Gly-L-Leu-Aib-L-Pro-L-Leu-Aib-D-Iva-L-Gln-L-Leuol

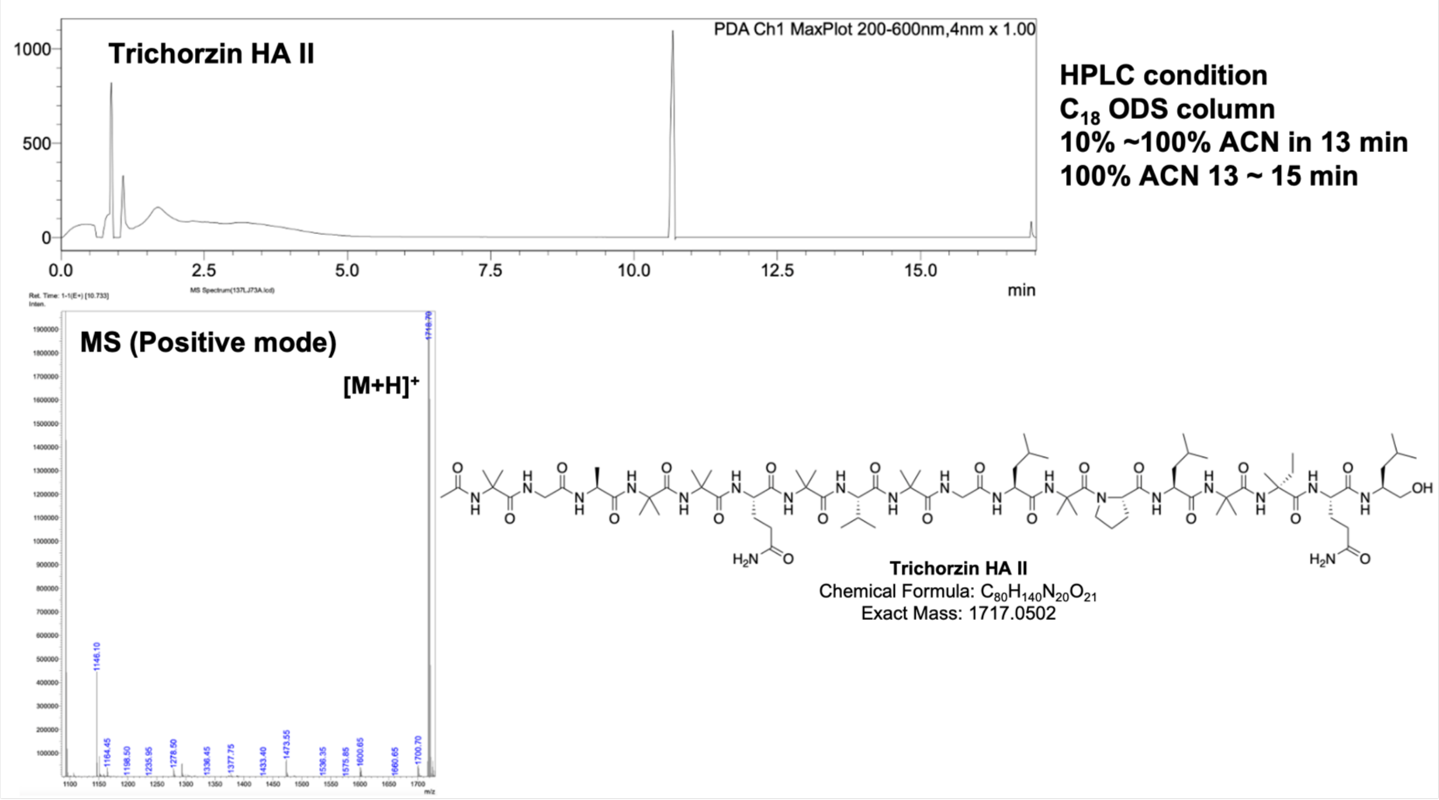

UPLC-MS spectra of trichorzin HA II

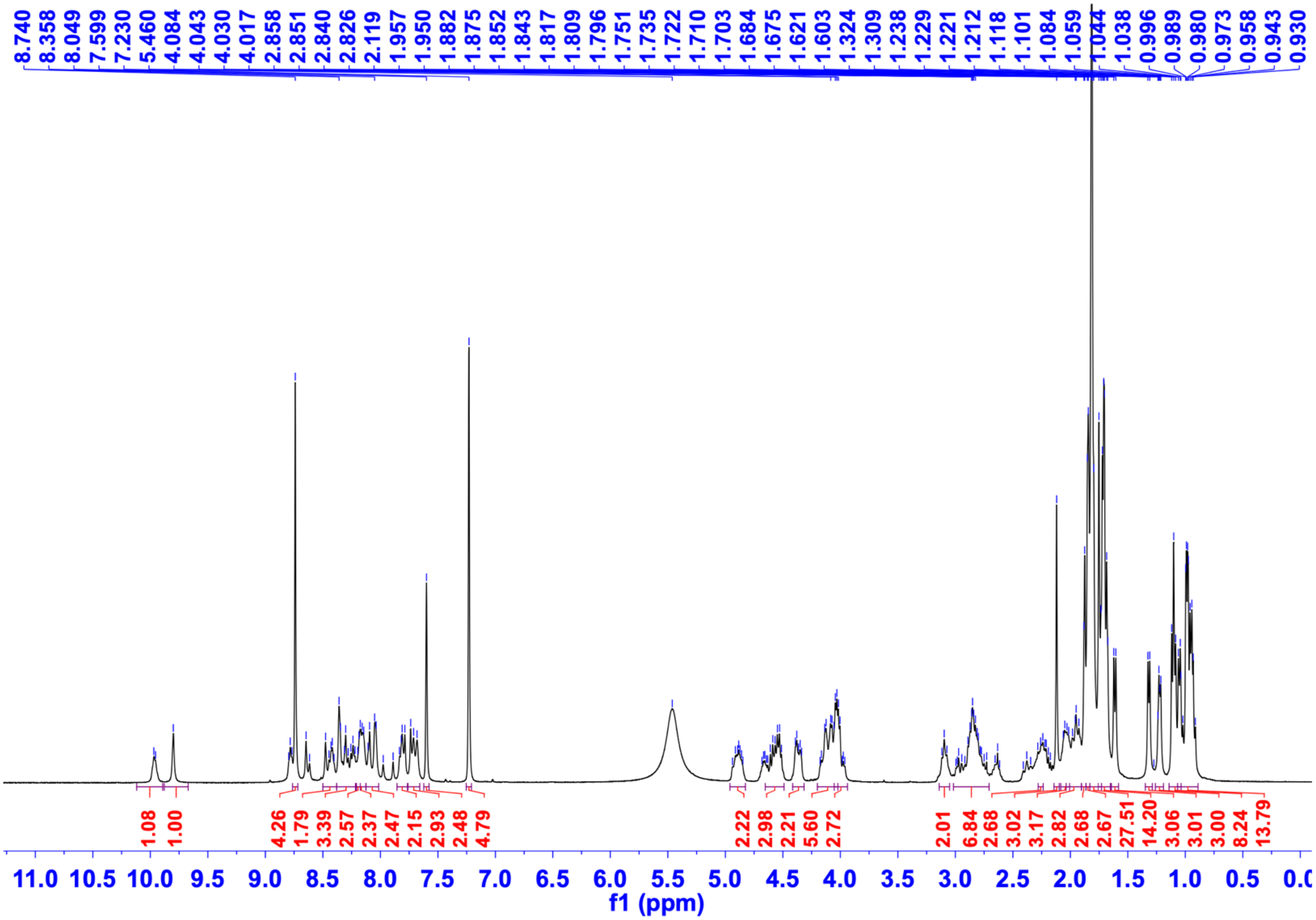

^{1}H NMR spectra of trichorzin HA II (400 MHz, pyridine-d_5)

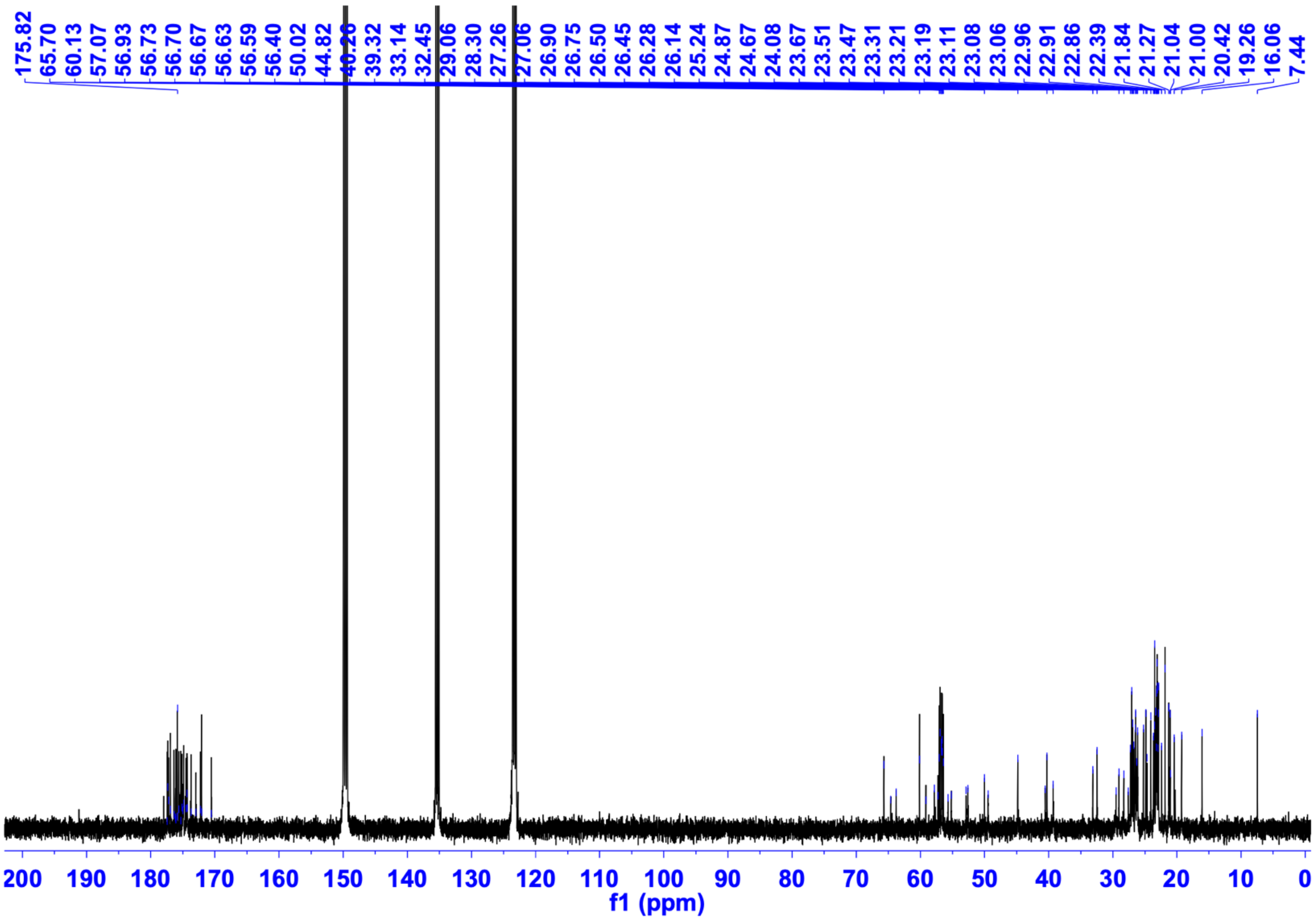

^{13}C NMR spectra of trichorzin HA II (100 MHz, pyridine-d_5

Ac-D-Iva-L-Gln-L-Leu-L-Ile-Aib-L-Pro-L-Iva-L-Leu-Aib-L-Pro-L-Leuol

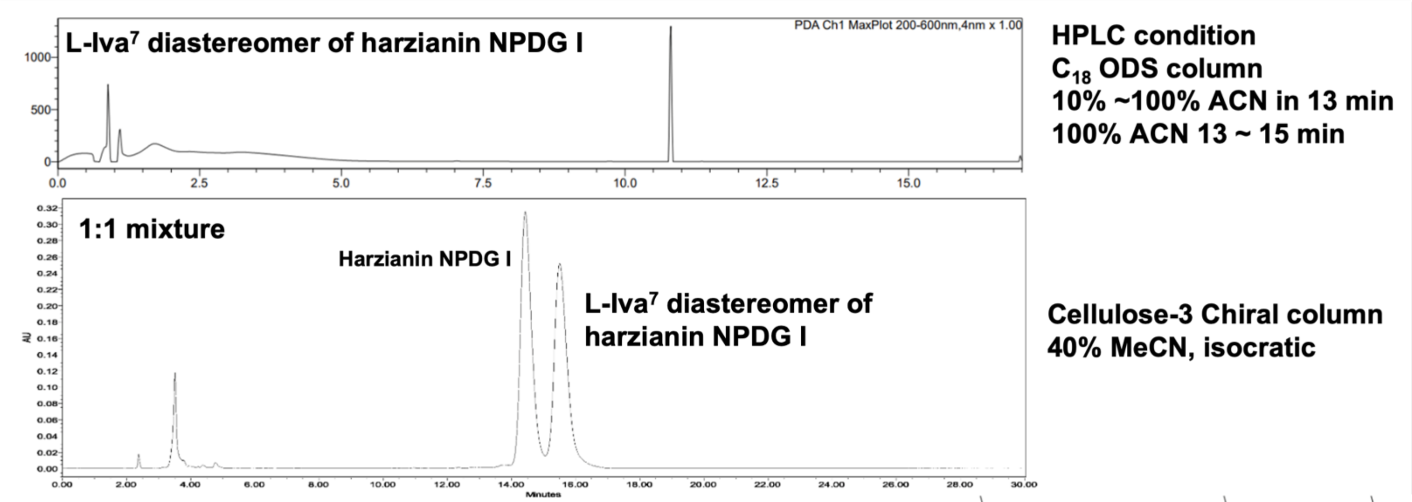

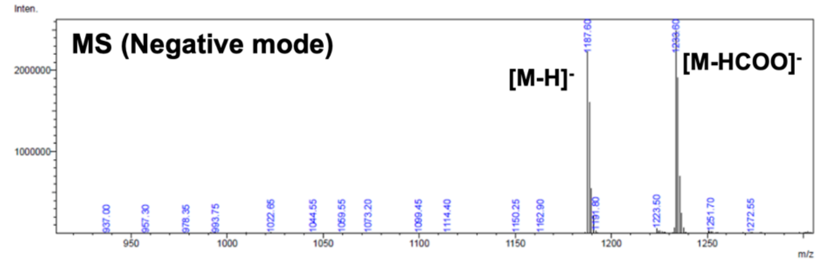

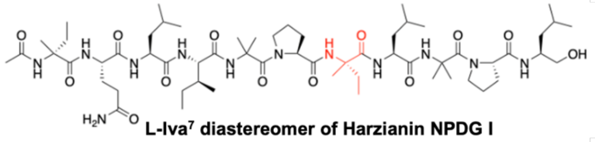

Chemical Formula: $C_{59}H_{104}N_{12}O_{13}$
Exact Mass: 1188.7846

Dd2 EC_{50} (μM)	3D7 EC_{50} (μM)	HepG2 EC_{50} (μM)
0.23 ± 0.03	0.21 ± 0.02	> 25

UPLC-MS spectra of L-Iva^{7} diastereomer of harzianin NPDG I

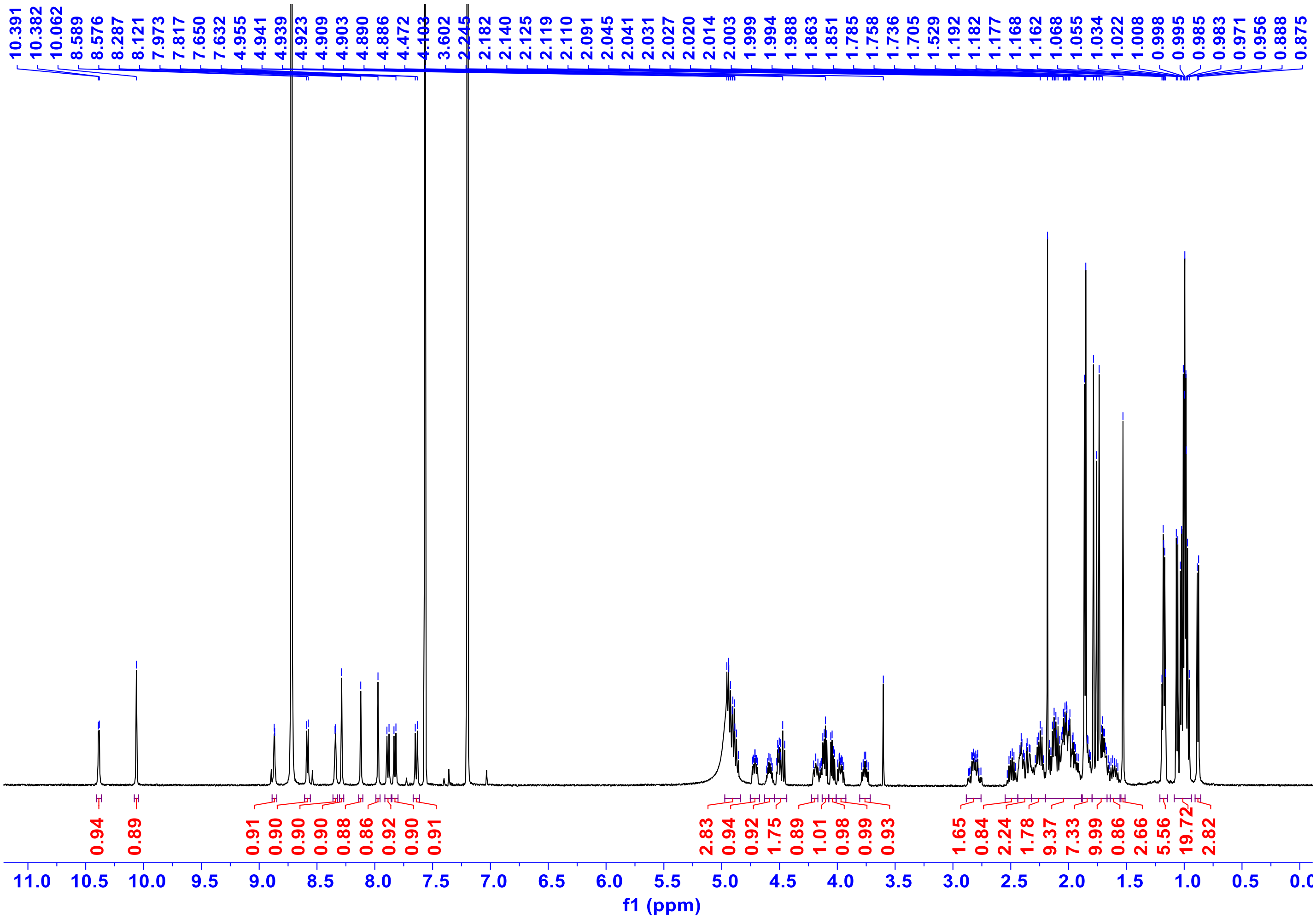

^{1}H NMR spectra of L-Iva[7] diastereomer of harzianin NPDG I (500 MHz, pyridine-d_5)

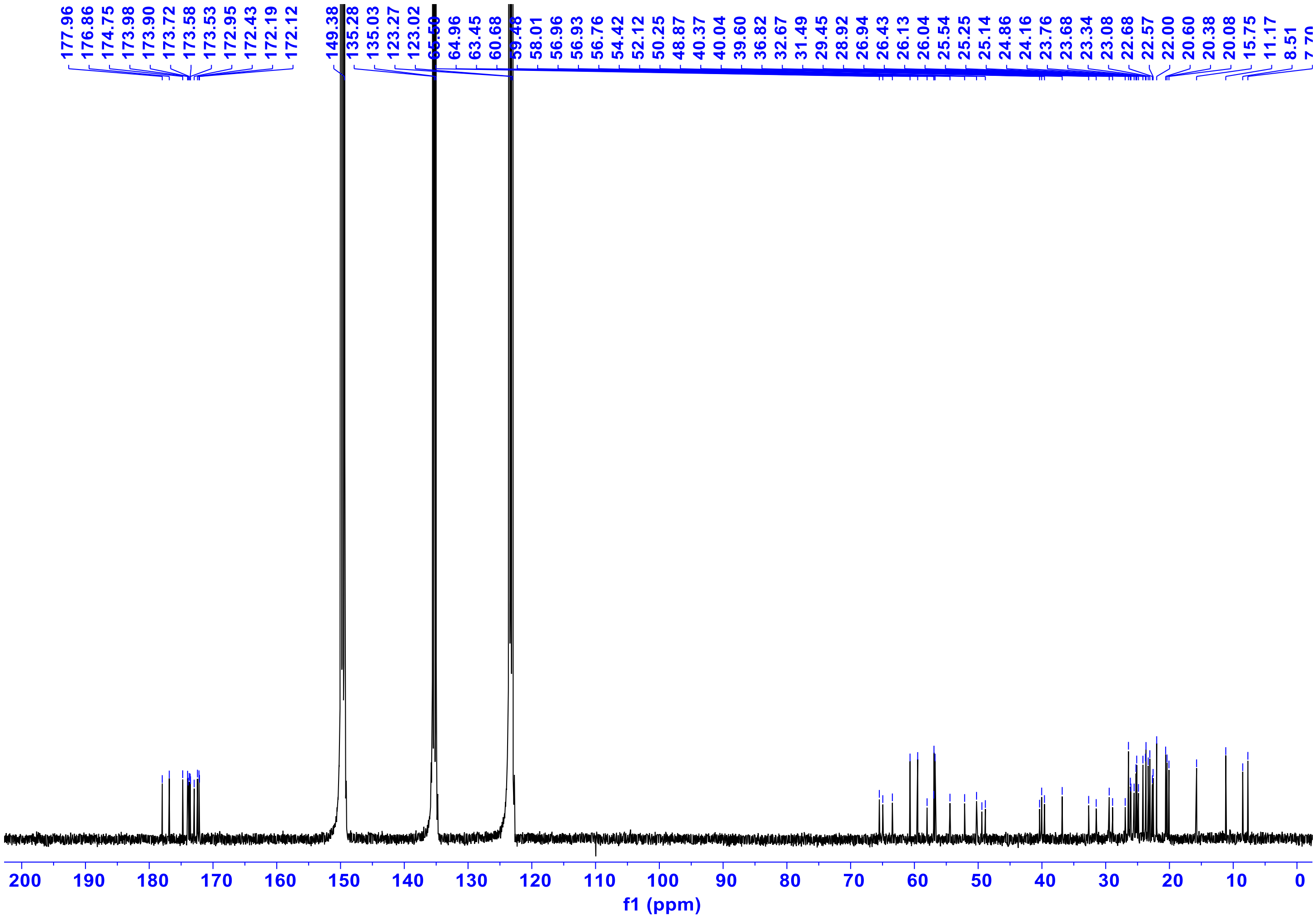

^{13}C NMR spectra of L-Iva^{7} diastereomer of harzianin NPDG I (100 MHz, pyridine-d_5)

Ac-Aib-L-Asn-L-Leu-L-Ile-Aib-L-Pro-D-Iva-L-Leu-Aib-L-Pro-L-Leu-NH_2

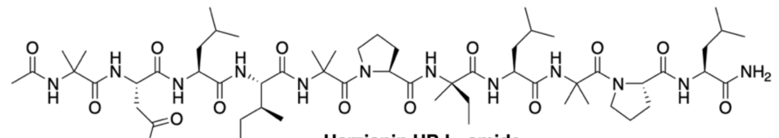

Harzianin HB I - amide
Chemical Formula: $C_{57}H_{99}N_{13}O_{13}$
Exact Mass: 1173.7485

Dd2 EC_{50} (μM)	3D7 EC_{50} (μM)	HepG2 EC_{50} (μM)
1.69 ± 0.01	2.64 ± 0.42	> 25

HPLC condition
C_{18} ODS column
10% ~100% ACN in 13 min
100% ACN 13 ~ 15 min

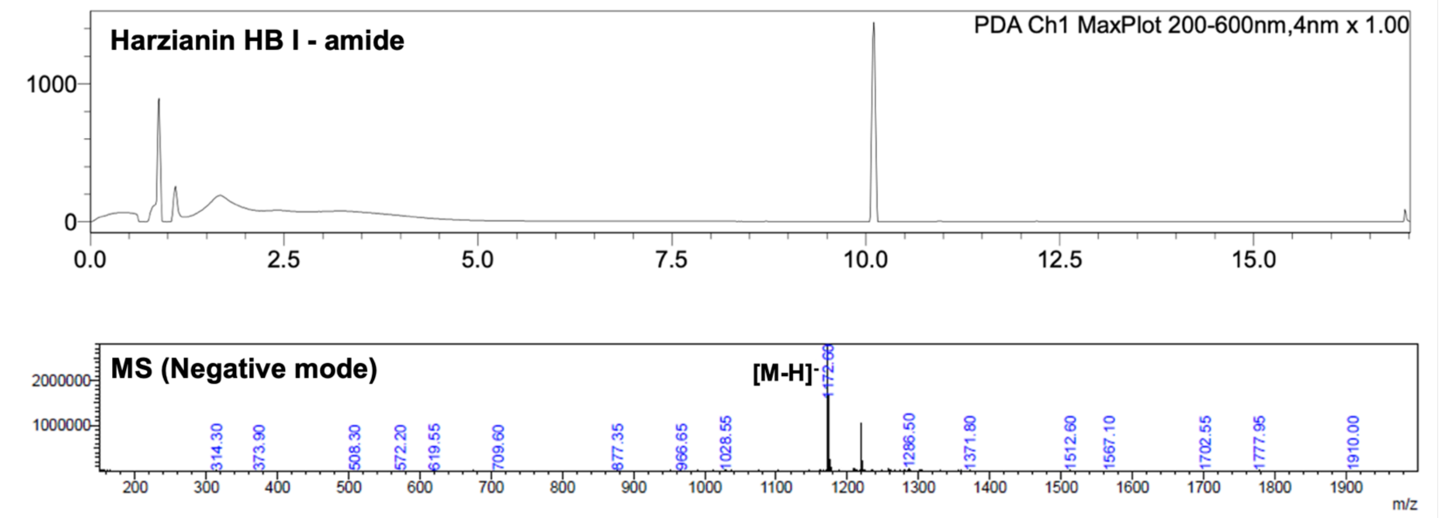

UPLC-MS spectra of harzianin HB I – amide

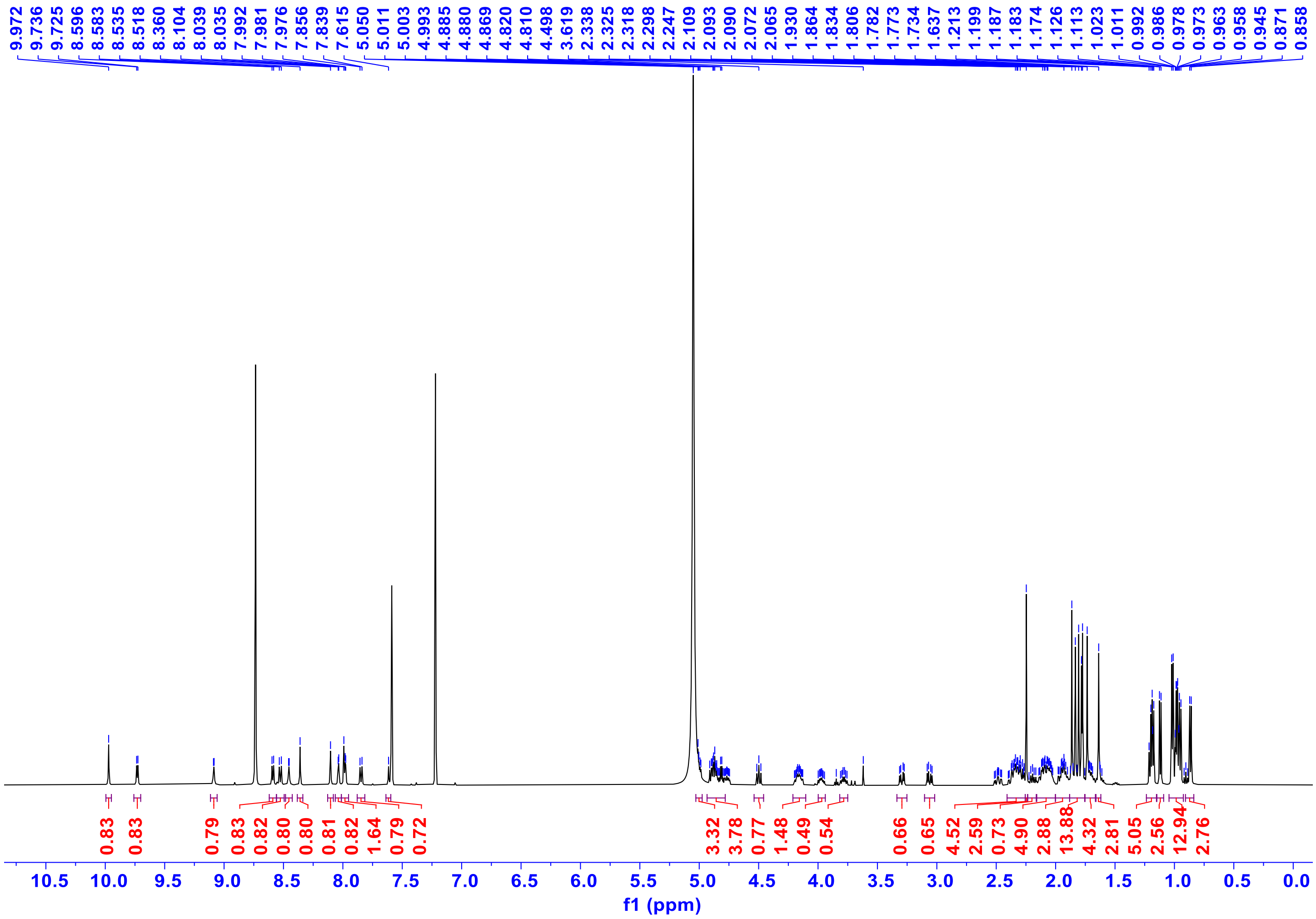

^{1}H NMR spectra of harzianin HB I – amide (500 MHz, pyridine-d_5)

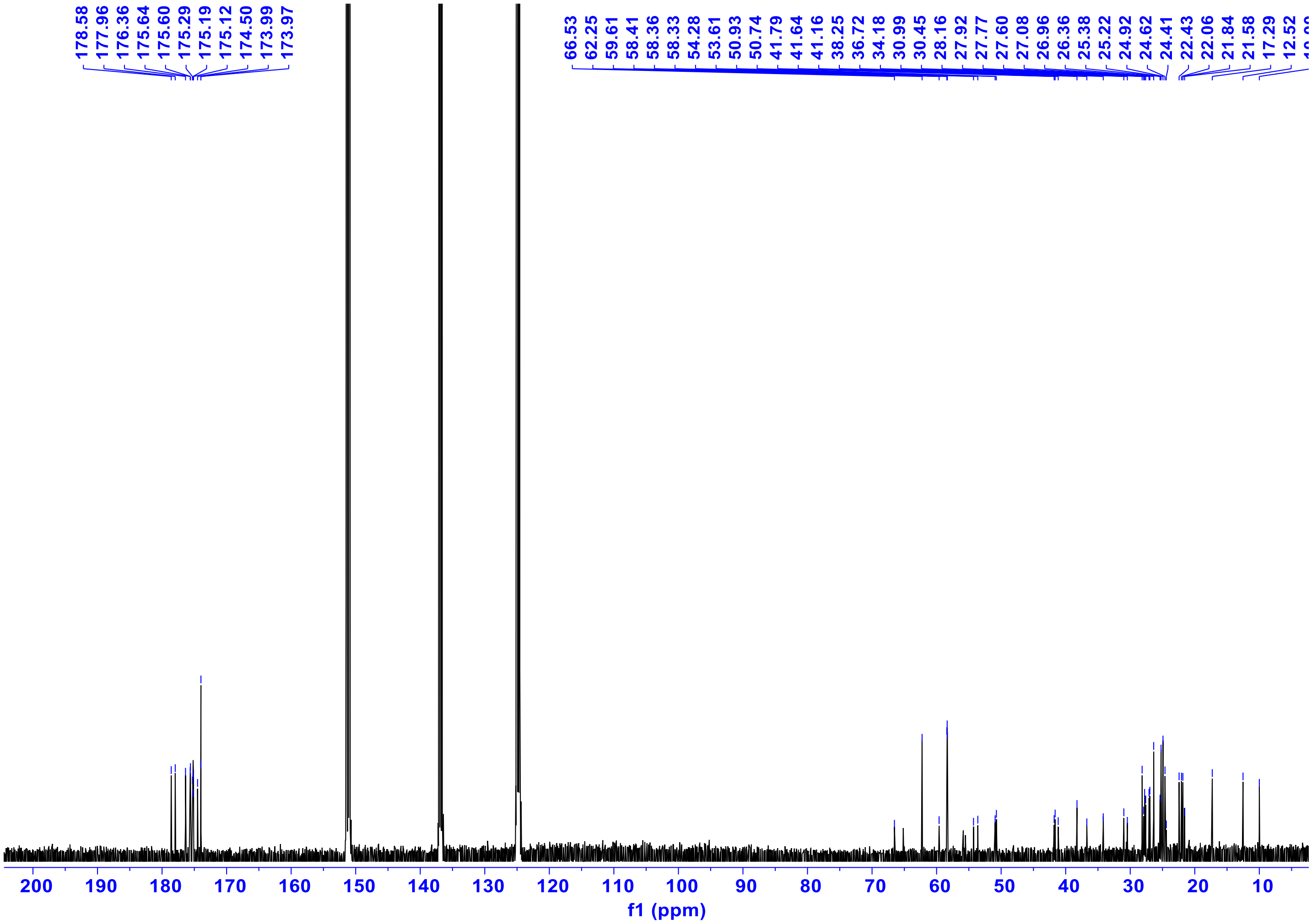

^{13}C NMR spectra of harzianin HB I – amide (100 MHz, pyridine-d_5)

Ac-D-Iva-L-Gln-L-Leu-L-Ile-Aib-L-Pro-D-Iva-L-Leu-Aib-L-Pro-L-Leu-NH_2

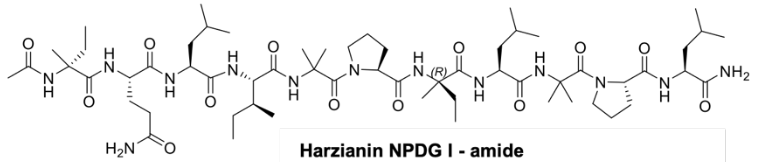

Harzianin NPDG I - amide
Chemical Formula: $C_{59}H_{103}N_{13}O_{13}$
Exact Mass: 1201.7798

Dd2 EC_{50} (μM)	3D7 EC_{50} (μM)	HepG2 EC_{50} (μM)
0.15 ± 0.03	0.18 ± 0.04	> 25

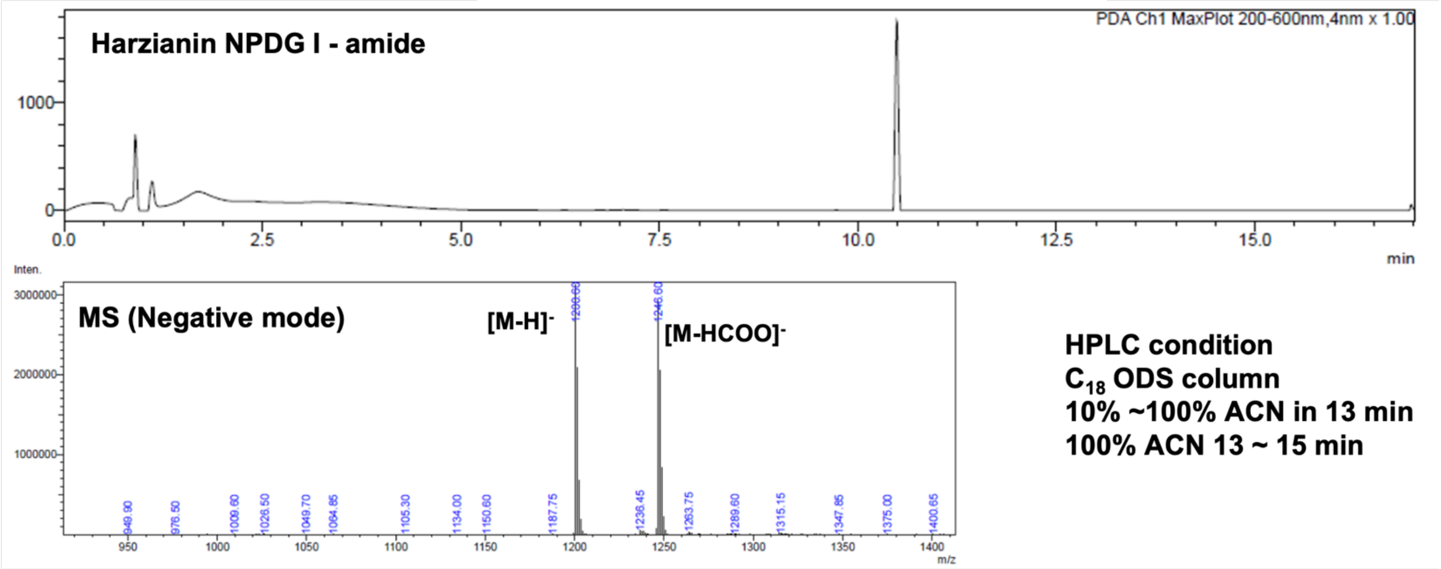

UPLC-MS spectra of harzianin NPDG I – amide

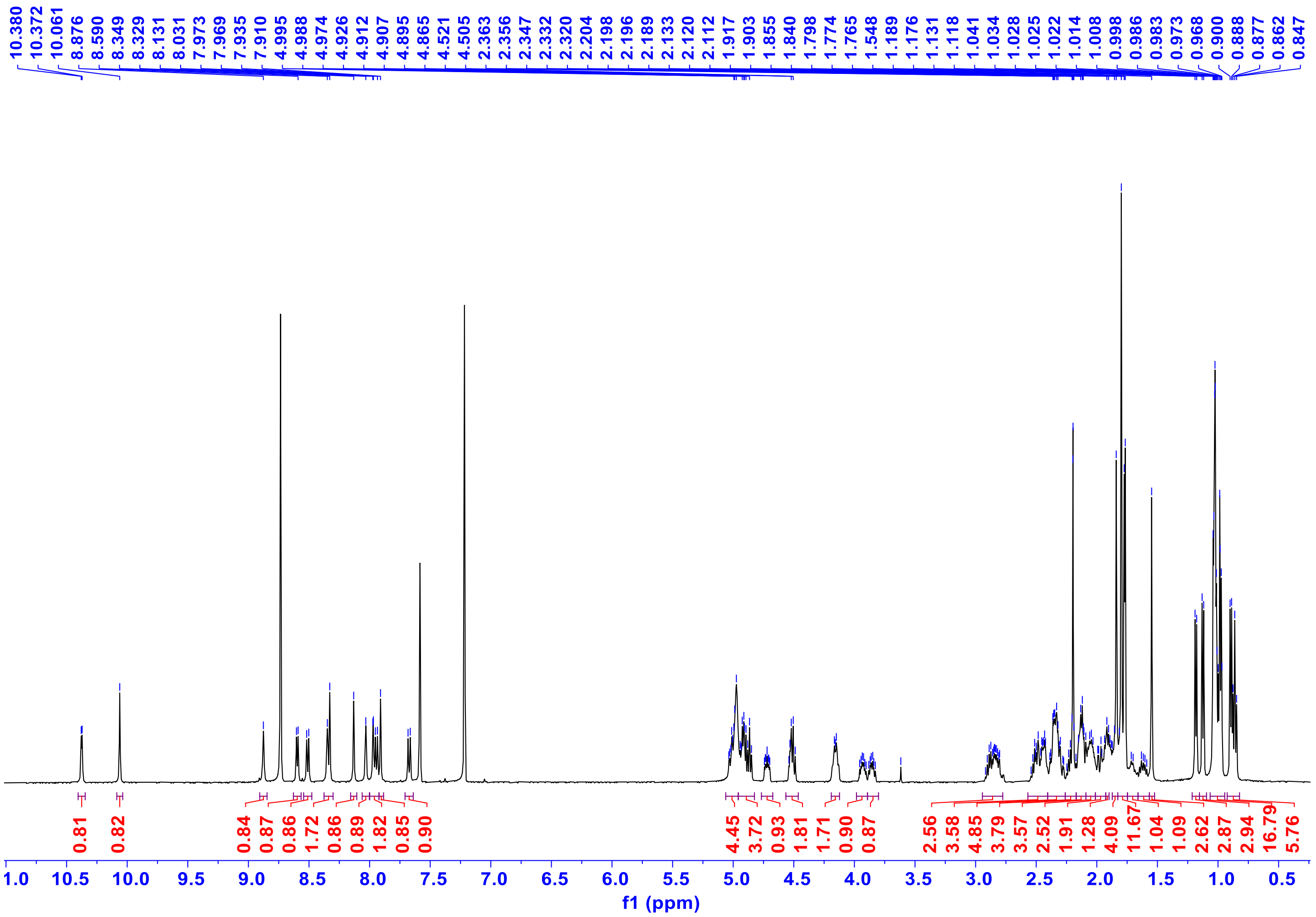

^{1}H NMR spectra of harzianin NPDG I – amide (500 MHz, pyridine-d_5)

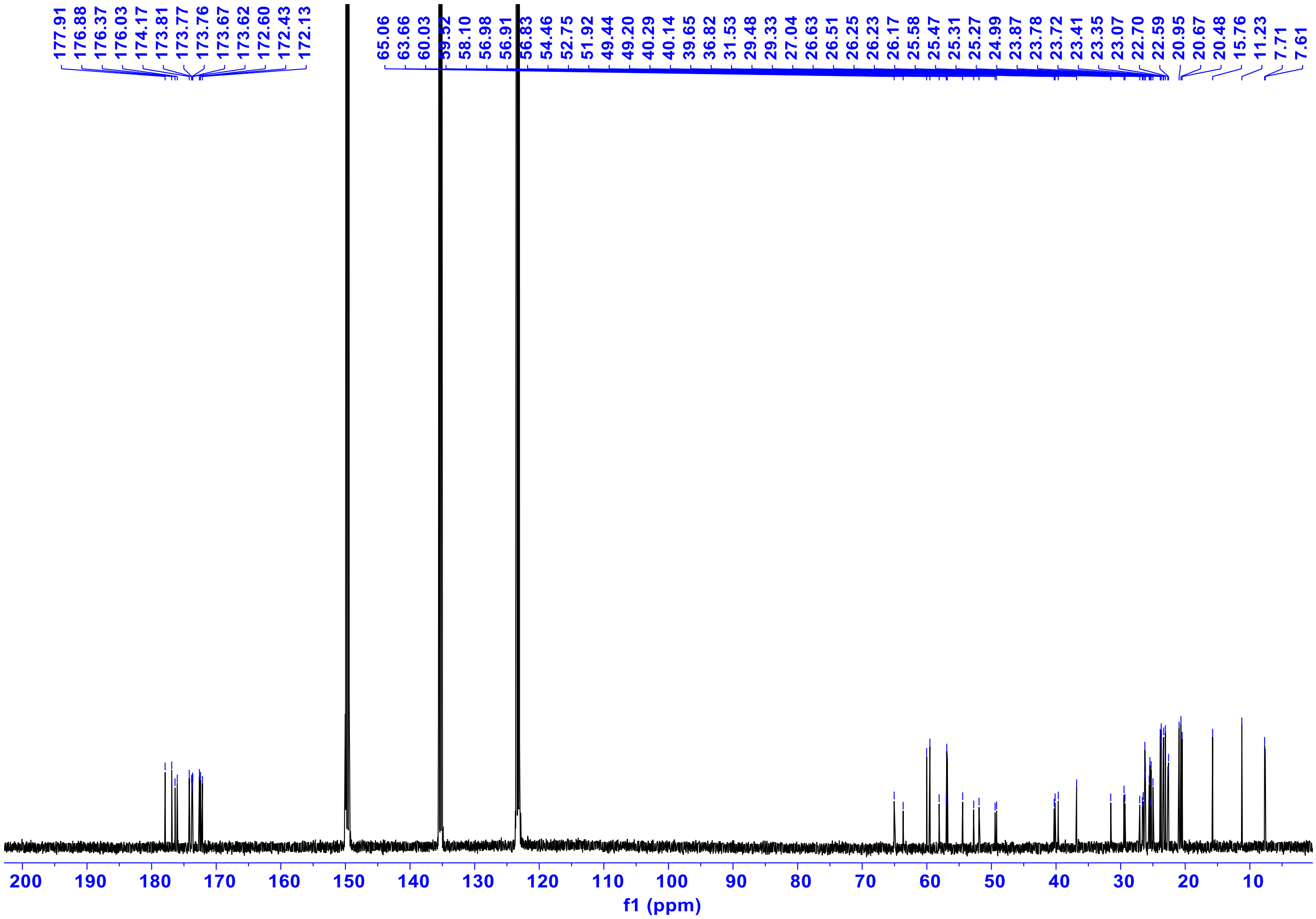

^{13}C NMR spectra of harzianin NPDG I – amide (100 MHz, pyridine-d_5)

Purity information of Alamethicin from Abcam

Alamethicin, Peptide antibiotic (ab141893)

Ac-Aib-Pro-Aib-Ala-Aib-Ala-Gln-Aib-Val-Aib-Gly-Leu-Aib-Pro-Val-Aib-Aib-Glu-Gln-Phl

Aib = 2-aminoisobutyric acid

Key features and details

- Peptide antibiotic
- CAS Number: 27061-78-5
- Purity: > 98%
- Soluble in DMSO to 10 mM
- Form / State: Solid
- Source: Trichoderma viride
